# RcbHLH59-RcPRs module enhances salinity stress tolerance by balancing Na^+^/K^+^ through callose deposition in rose (*Rosa chinensis*)

**DOI:** 10.1093/hr/uhac291

**Published:** 2022-12-30

**Authors:** Lin Su, Yichang Zhang, Shuang Yu, Lifang Geng, Shang Lin, Lin Ouyang, Xinqiang Jiang

**Affiliations:** College of Landscape Architecture and Forestry, Qingdao Agricultural University, Qingdao, 266000, China; College of Landscape Architecture and Forestry, Qingdao Agricultural University, Qingdao, 266000, China; College of Landscape Architecture and Forestry, Qingdao Agricultural University, Qingdao, 266000, China; College of Landscape Architecture and Forestry, Qingdao Agricultural University, Qingdao, 266000, China; College of Landscape Architecture and Forestry, Qingdao Agricultural University, Qingdao, 266000, China; Institute of Urban Agriculture, Chinese Academy of Agricultural Sciences, Chengdu, 610000, China; College of Landscape Architecture and Forestry, Qingdao Agricultural University, Qingdao, 266000, China

## Abstract

Basic helix–loop–helix (bHLH) proteins play pivotal roles in plant growth, development, and stress responses. However, the molecular and functional properties of bHLHs have not been fully characterized. In this study, a novel XI subgroup of the bHLH protein gene *RcbHLH59* was isolated and identified in rose (*Rosa* sp*.*). This gene was induced by salinity stress in both rose leaves and roots, and functioned as a transactivator. Accordingly, silencing *RcbHLH59* affected the antioxidant system, Na ^+^/K ^+^ balance, and photosynthetic system, thereby reducing salt tolerance, while the transient overexpression of *RcbHLH59* improved salinity stress tolerance. Additionally, RcbLHLH59 was found to regulate the expression of sets of pathogenesis-related (*PR*) genes in *RcbHLH59*-silenced (TRV-*RcbHLH59*) and *RcbHLH59*-overexpressing (*RcbHLH59-*OE) rose plants. The *RcPR4/1* and *RcPR5/1* transcript levels showed opposite changes in the TRV-*RcbHLH59* and *RcbHLH59-*OE lines, suggesting that these two genes are regulated by *RcbHLH59*. Further analysis revealed that RcbHLH59 binds to the promoters of RcPR4/1 and RcPR5/1, and that the silencing of *RcPR4/1* or *RcPR5/1* led to decreased tolerance to salinity stress. Moreover, callose degradation- and deposition-related genes were impaired in *RcPR4/1*- or *RcPR5/1*-silenced plants, which displayed a salt tolerance phenotype by balancing the Na^+^/K^+^ ratio through callose deposition. Collectively, our data highlight a new RcbLHLH59-RcPRs module that positively regulates salinity stress tolerance by balancing Na^+^/K^+^ and through callose deposition in rose plants.

## Introduction

Immovable plants are frequently affected by environmental stress during their life cycle. Salinization is a universal environmental problem that affects plant growth and yield [1]. Approximately 20% of the world’s irrigated farmland is adversely affected by soil salinization [2]. In this context, the breeding and development of salt-tolerant plant varieties is critical to increase plant productivity and yield. Meanwhile, plants have evolved complex systems at the physiological or molecular level to adapt to salinity stress. These strategies include osmotic regulation, cytosolic ion and redox capacity, and efficient stomatal operation [3]. Transcription factors (TFs) play an important role in gene regulation, transcription, and signal transmission under salinity stress. In previous studies, various categories of TFs have been found to be widely involved in the response to salinity stress, such as NACs [4, 5], WRKYs [6, 7], MYBs [8, 9], bZIPs [10, 11], and bHLHs [12, 13].

In particular, the basic helix–loop–helixes (bHLHs) are a superfamily of TFs with DNA-binding and dimerization capabilities. They contain the conserved bHLH domain, comprising ~60 amino acids with two functionally distinct regions, the basic region and the helix–loop–helix (HLH) [14], The basic region functions as a DNA-binding motif, while the HLH region acts as a dimerization domain and allows the formation of homodimers or heterodimers [15]. According to their phylogenetic relationships, DNA-binding motifs, and functional properties, bHLHs can be divided into six main groups containing 45 subfamilies [16]. In addition, bHLHs play distinct roles in plant growth, development, and stress response. The role of bHLHs in salinity stress has been studied in model plant species, such as *Arabidopsis* [17, 18] and rice [19, 20]. For example, in *Arabidopsis* the bHLH TF MYC2 improves salt tolerance by regulating the delta1-pyrroline-5-carboxylic acid synthase 1 (*P5CS1*) gene, which in turn regulates proline synthesis under salt stress [21]. In rice, the bHLH TF BEAR1 is induced by salt stress and regulates salt responses by controlling ion transporters and salt-responsive marker genes [22]. Pepper bHLH TF CabHLH035 increases salt tolerance by modulating Na^+^/K^+^ and proline biosynthesis [23]. In ornamental plants, bHLHs are mainly involved in the anthocyanin and abiotic stress responses. *Chimonanthus praecox* CpbHLH1 inhibits anthocyanin accumulation in transgenic *Arabidopsis* and tobacco [24]. AabHLH35 from *Anthurium andraeanum* confers cold and drought tolerance [25]. Overexpressing the *Zoysia japonica* bHLH gene, *ZjICE2*, in *Arabidopsis* enhances reactive oxygen species (ROS) scavenging and confers salt stress tolerance [26]. However, the functions and molecular mechanisms of the bHLHs involved in salinity stress in ornamental crops remain unclear.

In addition to TFs, many functional proteins are upregulated in response to adverse environmental stimuli. In particular, pathogenesis-related proteins (PRs) are upregulated in response to various biotic and abiotic stressors [27]. Currently, PRs are classified into 17 families according to their order of discovery, and exist widely in various plant organs [28]. Evidence indicates that PRs exert powerful roles to cope with various unfavorable environments [29, 30]. At present, most abiotic stress studies on PRs have focused on drought stress [31, 32], while little is known about their effects on salt stress.

Cell wall integrity is an important index for determining plant growth and for coping with salt stress. Accumulated Na^+^ in the apoplast binds directly to cell wall components and affects their chemistry, thereby disrupting cell wall integrity [3]. The main components of plant cell walls are cellulose, hemicellulose, pectin, and several glycoproteins [3]. Callose is a (1,3)-β-glucan, a cell wall polymer, with a composition similar to that of cellulose. Under unfavorable conditions, callose accumulates in the secondary cell wall, which plays a major role in maintaining the mechanical stability of the cell wall [33]. Simultaneously, the accumulation of callose prevents the penetration of harmful ions and protects internal tissues [34]. Callose accumulation has been implicated in adverse stresses, such as disease, heavy metal stress, and drought and salt stress [35–38]. In recent years, many genes have been reported to be involved in callose accumulation, such as *PR2* [39] and *callose synthase* (*Cals*) [40]. Under salinity stress, *Arabidopsis* cysteine-rich receptor-like kinase 2 (CRK2) interacts with CALS1 and induces callose accumulation, thereby increasing salt tolerance [41]. However, the molecular mechanisms underlying callose deposition in response to salinity stress remain largely unclear. Thus, there is a need to study the mechanism of callose deposition and stress responsiveness to identify the key bHLH and PRs involved in these processes.

Rose (*Rosa* spp*.*) is an important commercial flower crop of the Rosaceae family that is grown worldwide [42]. Roses are often affected by a variety of adverse environmental stimuli, wherein salinity stress is one of the main factors that limit their characteristics and productivity, especially in saline-alkali regions. In addition, salt stress often leads to poor growth, as well as the aggravation of diseases or insect pests in many rose varieties [43]. Thus, improving our understanding of the molecular mechanisms underlying salt stress in roses is particularly important. In the present study we analyzed and identified *RcbHLH59*, which is highly expressed in the leaves of roses under salinity stress. Silencing *RcbHLH59* was found to decrease salinity tolerance, whereas *RcbHLH59* overexpression improved salinity tolerance. Meanwhile, *RcbHLH59* was found to directly bind to the promoters of *RcPR4/1* and *RcPR5/1*, while the silencing of *RcPR4/1* or *RcPR5/1* affected the accumulation and composition of callose in rose leaves. These results highlight the role of a new *RcbHLH59-RcPRs* module in enhancing salinity stress by balancing Na^+^/K^+^ through callose composition and degradation.

## Results

### Identification of *RcbHLH59*

**Figure 1:**
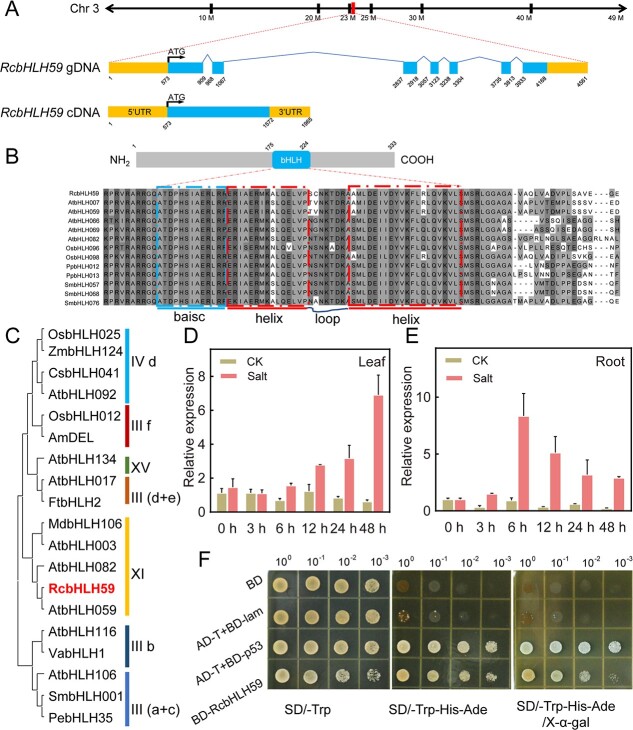
*RcbHLH59* is a salt-induced transcriptional activator. (A) The genomic sequence of *RcbHLH59* spans 3634 bp and consists of six exons. The full-length cDNA of *RcbHLH59* contains an ORF of 999 bp. (B) Domain structure and sequence alignment of RcbHLH59 with other plant bHLH proteins. The red line segments represent the two helix domains. Identical and similar residues are shown in black and gray, respectively. (C) Phylogenetic tree of RcbHLH59 and to other plant bHLH proteins. GenBank accession numbers are as follows: *AtbHLH3* (At4g16430), *AtbHLH17* (At2g46510), *AtbHLH59* (At4g02590), *AtbHLH82* (At5g58010), *AtbHLH92* (At5g43650), *AtbHLH106* (At2g41130), *AtbHLH122* (At1g51140), *AtbHLH134* (At5g15160), *ZmbHLH124* (GRMZM2G132550), *MdbHLH130* (MDP0000581816), *PebHLH35* (KJ363186.1), *CsbHLH041* (Solyc07g039570.2.1), *AmDEL* (AAA32663.1), *VabHLH1* (JQ911779.1), *FtbHLH2* (AMK74868.1), *SmbHLH001*, *OsbHLH012* (LOC_Os01g39480), *OsbHLH025* (LOC_Os01g09990), *RcbHLH59* (RcHm_v2.0_Chr3g0451111). The neighbor-joining method in MEGA X was utilized to construct the phylogenetic tree with 2000 bootstrap replicates. Different colors represent different groups. (D, E) Expression profiles of *RcbHLH59* in rose (D) leaves and (E) roots under 200 mM NaCl. Error bars indicate standard deviations (SDs) based on three biological replicates. (F) Transactivation activity assay in yeast cells. Yeast strains that contained ‘empty’ BD plasmids and were co-transformed with pGADT7-T (AD-T) and pGBKT7-p53 (BD-p53) were used as a positive control; yeast strains co-transfected with AD-T and pGBKT7-lam (BD-lam) were used as negative controls. Yeast cells co-transformed with different vectors were grown on SD/−Trp and SD/−Trp−His−Ade with and without X-α-Gal supplementation to examine the transcriptional activation activity of *RcbHLH59*. 10^0^, 10^−1^, 10^−2^, and 10^−3^ represent the original concentration (OD_600_ = 0.2) and 10-, 100-, and 1000-fold dilutions of yeast, respectively.

Based on previous studies [44], a differentially expressed gene was found to be induced by both drought and salinity stress (Supplementary Data Fig. S1). The protein encoded by *RcbHLH59* shared good similarity (55.29%) with *Arabidopsis* AtbHLH59 (Supplementary Data Fig. S1); this gene was denoted as *RcbHLH59*. Next, specific primers (Supplementary Data Table S1) were used to amplify *RcbHLH59*, followed by alignment using the rose database. The corresponding gDNA sequence of *RcbHLH59* was 3634 bp in length and had seven exons and six introns, which was mapped to chromosome 3 of *Rosa chinensis* ‘Old Blush’. The length of *RcbHLH59* cDNA was 1965 bp with a 999-bp potential open reading frame (ORF), 573-bp 5′ untranslated region (UTR), and 393-bp 3′ UTR, which encoded 333 amino acids, with a weight (Mw) of 35.3 kDa and an isoelectric point (pI) of 5.81 (Fig. 1A). The alignment of RcbHLH59 with its homologs derived from four plant species (*Arabidopsis thaliana*, *Oryza sativa*, *Physcomitrella patens*, and *Selaginella moellendorffii*) showed that both possessed a conserved bHLH domain, located at 175–224 amino acids in the sequence (Fig. 1B). Next, 19 bHLH proteins were collected from 10 plant species, including two monocots (*Zea mays* and *O. sativa*) and eight dicotyledons (*A. thaliana*, *Malus domestica*, *Populus euphratica*, *Cucumis sativus*, *Antirrhinum majus*, *Vitis vinifera*, *Fagopyrum tataricum*, and *S. moellendorffii*), to create a phylogenetic tree. The results showed that RcbHLH59 and other bHLH proteins consisted of seven individual groups: groups III (a + c), III b, III (d + e), III f, IV d, XI, and XV. RcbHLH59, together with AtbHLH59 and AtbHLH82, belonged to subgroup XI (Fig. 1C, Supplementary Data Table S2).

### Salinity stress induces the transactivator *RcbHLH59*

The expression profiles of RcbHLH59 were determined in a time-course of salinity stress in the leaves and roots of rose plants. Compared with the control treatment at 0 hours, *RcbHLH59* expression gradually increased at 6 hours (1.57-fold), 12 hours (2.79-fold), and 24 hours (3.17-fold), reaching a peak of ~6.92-fold at 48 hours (Fig. 1D). In rose roots, *RcbHLH59* expression peaked at ~8.35-fold at 6 hours, then decreased at 12 hours (5.12-fold), 24 hours (3.18-fold) and 48 hours (2.90-fold) (Fig. 1E). This result is in accordance with previous transcriptomic analyses of plants under salinity stress (Supplementary Data Fig. S1). Next, we determined the expression levels of *RcbHLH59* under drought and abscisic acid (ABA) treatments (Supplementary Data Fig. S2). The results showed that the expression of *RcbHLH59* was upregulated, peaking after 24 hours of drought stress. However, the expression was not obviously changed under ABA treatment for 12, 24, and 48 hours. ABA treatment for 72 hours increased the *RcbHLH59* expression level by ~2.05-fold compared with 0 hours (Supplementary Data Fig. S2B). To evaluate the transactivation activity of RcbHLH59, full-length RcbHLH59 was fused to the GAL4 DNA-binding domain (BD) to construct BD-RcbHLH59. BD-RcbHLH59 and the positive control grew well on SD media lacking tryptophan (Trp), adenylate (Ade), and histidine (His) supplemented with X-α-Gal (Fig. 1F), suggesting that RcbHLH59 has transcriptional activation activity. These results indicate that RcbHLH59 is a salt-induced transcription activator.

### Silencing of *RcbHLH59* in rose decreases salinity tolerance

**Figure 2:**
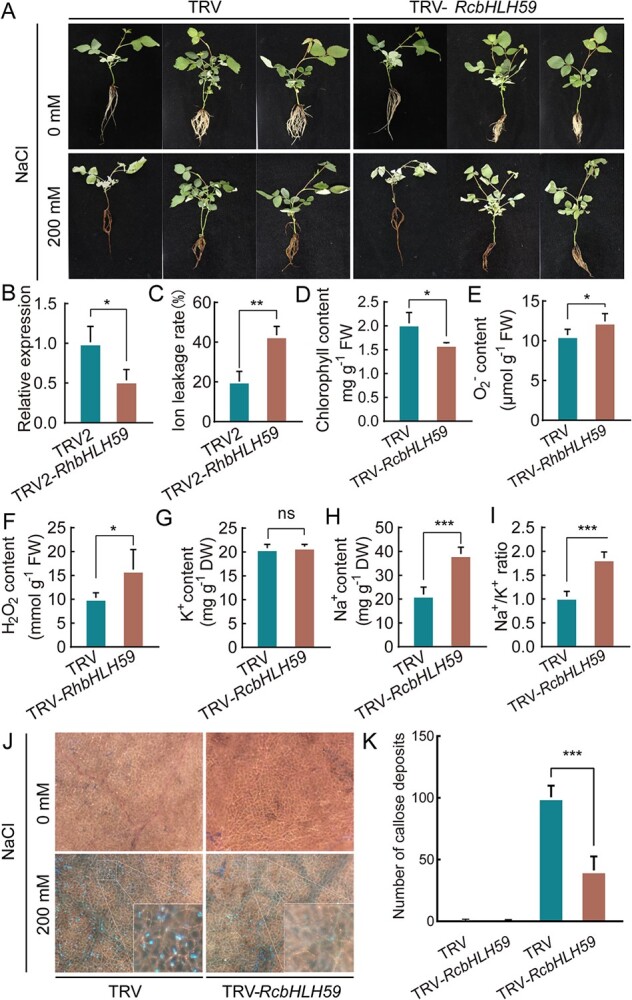
Silencing *RcbHLH59* decreases salt tolerance in rose. (A) Phenotype of TRV and TRV-*RcbHLH59* rose plants under 200 mM NaCl treatment for 3 days. (B) Relative expression of *RcbHLH59* in TRV and TRV-*RcbHLH59*. Relative expression levels refer to the expression value of *RcbHLH59* in relation to *RcUBI2* (internal reference gene). (C) Ion leakage rate, (D) chlorophyll content, (E) O_2_^−^ content, (F) H_2_O_2_ content, (G) K^+^ content, (H) Na^+^ content, and (I) Na^+^/K^+^ ratio between TRV and TRV-*RcbHLH59* leaves under 200 mM NaCl treatment for 3 days. (J) Epidermis of TRV and TRV-*RcbHLH59* leaves under a fluorescence microscope, with blue–white spots of callosum. The white border is the insets with photographic enlargements. (K) Statistical results of the number of corpus callosums. Data are mean with standard deviation (*n* = 3) of at least three independent experiments. Statistically significant differences (ns, *P* > .05; ^*^, *P* ≤ .05; ^**^, *P* ≤ .01; ^***^, *P* ≤ .001) were determined by *t*-test.

To determine the role of *RcbHLH59*, the gene was silenced in rose plants using a virus-induced gene silencing (VIGS) system and the role of *RcbHLH59* in response to salt stress was investigated using control (TRV) and *RcbHLH59*-silenced (TRV-*RcbHLH59*) rose plants (Fig. 2). No obvious phenotypes were found in either TRV or TRV-*RcbHLH59* under normal growth conditions. By contrast, under 200 mM NaCl treatment, the TRV-*RcbHLH59* plants exhibited more chlorosis and dryness, and less root formation than the TRV controls (Fig. 2A). RT–qPCR analysis confirmed that *RcbHLH59* in TRV-*RcbHLH59* exhibited a reduced expression level of ~50% compared with the TRV control (Fig. 2B). The chlorophyll content in TRV-*RcbHLH59* was lower than that in the TRV control (Fig. 2D). Moreover, the ion leakage rate (Fig. 2C) and the superoxide anion (O_2_^−^) (Fig. 2E) and hydrogen peroxide (H_2_O_2_) content (Fig. 2F) in TRV-*RcbHLH59* were significantly higher than those in TRV, implying that the production of ROS or impaired ROS detoxification leads to altered oxidative damage when silencing *RcbHLH59* under salinity stress. We also measured the content of Na^+^ and K^+^ in TRV and TRV-*RcbHLH59* (Fig. 2G–I) and found no significant difference in K^+^ content between TRV and TRV-*RcbHLH59* (Fig. 2G), while the Na^+^ content in TRV was 21.9 mg/g, which was significantly lower than that in TRV-*RcbHLH59* (38.9 mg/g) (Fig. 2H). Furthermore, Na^+^/K^+^ balance was found to be a key factor under salinity stress. The Na^+^/K^+^ ratio in TRV was 1.05, which was significantly higher than that of TRV-*RcbHLH59* (1.85) under salinity stress (Fig. 2I), implying that *RcbHLH59* influenced the Na^+^/K^+^ ratio. We also found that TRV-*RcbHLH59* accumulated fewer callose deposits than the TRV controls (Fig. 2J and K), indicating that *RcbHLH59* may influence callose deposition.

*F*_v_/*F*_m_ is the maximum potential quantum efficiency of photosystem II (PSII). The chlorophyll fluorescence parameters of both TRV and TRV-*RcbHLH59* were the same as in the above results. Under normal growth conditions, *F*_v_/*F*_m_ was maintained between 0.7 and 0.8. Under salinity stress conditions, the *F*_v_/*F*_m_ ratio in TRV-*RcbHLH59* was 25% lower than that in the TRV controls (Supplementary Data Fig. S3), indicating that stronger photoinhibition existed in TRV-*RcbHLH59*. In summary, these results indicate that the silencing of *RcbHLH59* affects the plant antioxidant system, Na^+^/K^+^ balance, and photosynthetic system, leading to decreased tolerance of salinity stress.

### *RcbHLH59* overexpression improves salinity tolerance in roses

**Figure 3:**
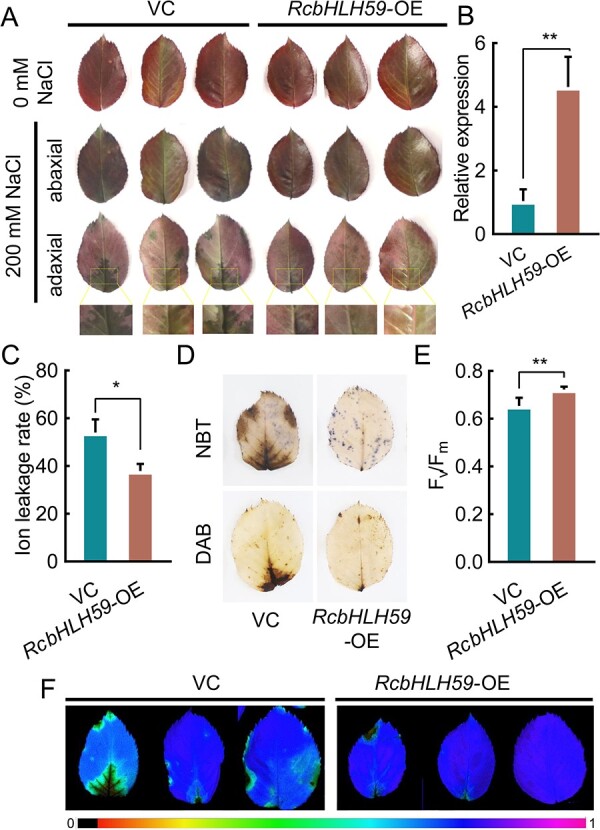
Overexpression of *RcbHLH59* improved tolerance to salinity stress. (A) Phenotype of VC and *RcbHLH59*-OE under salinity stress. Young tender rose leaves of the same size were used for transient overexpression with (*RcbHLH59*-OE) or without (VC) *RcbHLH59* under normal conditions and 200 mM NaCl treatment for 5 days. The yellow border is the insets with photographic enlargements. (B) Relative expression of *RcbHLH59* in VC and *RcbHLH59*-OE rose leaves. Relative expression levels refer to the expression value of *RcbHLH59* relative to *RcUBI2*. (C) Ion leakage rate between VC and *RcbHLH59*-OE leaves. (D) NBT and DAB staining of VC and *RcbHLH59*-OE leaves in response to salinity stress*.* (E) Quantification of chlorophyll imaging. (F) Chlorophyll fluorescence images of VC and *RcbHLH59*-OE rose leaves. Error bars represent values from minimum to maximum. Data are mean with standard deviation (*n* = 3) of at least three independent experiments, each with more than three plants. Statistically significant differences (^*^, *P* ≤ .05; ^**^, *P* ≤ .01) were determined by *t*-tests.

Next, we overexpressed *RcbHLH59* in rose leaves under salinity stress (Fig. 3). *RcbHLH59* was transiently overexpressed under a constitutive super-promoter (VC) to construct *RcbHLH59-*overexpressing (*RcbHLH59*-OE) plants. The *RcbHLH59* expression level in the *RcbHLH59*-OE plants was 4-fold higher than that in the VC controls (Fig. 3B). Under 0 mM NaCl conditions, we observed no phenotypic differences between VC and *RcbHLH59*-OE plants (Fig. 3A). However, after treatment with 200 mM NaCl for 5 days, more water spots accumulated on the bottom and margin of the adaxial end of the VC leaves, whereas the *RcbHLH59*-OE plants showed a less wilted phenotype. Furthermore, electrolyte leakage analysis showed that the leaves of *RcbHLH59*-OE plants exhibited a lower degree of damage during salinity stress than the leaves of the VC controls (Fig. 3C). We also analyzed the accumulation of ROS in VC and *RcbHLH59*-OE using 3,3-diaminobenzidine (DAB) and nitroblue tetrazolium (NBT) staining under 200 mM NaCl treatment. The *RcbHLH59*-OE accumulated less brown and blue color, with fewer speckles and less local tissue necrosis than the VC controls (Fig. 3D). Consistently, after 200 mM NaCl treatment, chlorophyll imaging systems indicated that the *F*_v_/*F*_m_ in *RcbHLH59*-OE plants was 0.73, which was significantly higher than that in the VC controls (0.65) (Fig. 3E). A more wilted leaf phenotype was observed in the VC controls than in *RcbHLH59*-OEs (Fig. 3F). These results indicate that the overexpression of *RcbHLH59* in rose plants leads to enhanced salinity tolerance.

### Pathogenesis-related genes are impaired in *RcbHLH59-*silenced and -overexpressing plants

**Figure 4:**
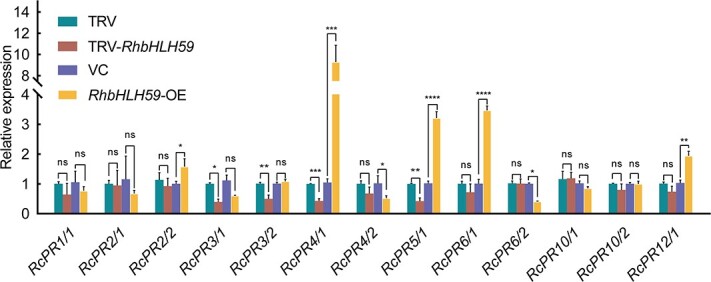
Expression analysis of 13 *pathogenesis-related* (*PR*) genes of *RcbHLH59*-silenced and *RcbHLH59*-overexpressing leaves under 200 mM NaCl treatment for 3 days. GenBank accession numbers of selected *PRs* are listed in Supplementary Data Table S3. *RcUBI2* was used as an internal control. Error bars indicate standard deviations based on six biological replicates. Statistically significant differences (ns, *P* > .5, ^*^, *P* ≤ .05; ^**^, *P* ≤ .01, ^***^, *P* ≤ .001, ^****^, *P* ≤ .001) were determined by *t*-tests.

In our previous studies, *RcbHLH59* interacted with *RcTLP6*, a *PR* family gene, and *RcTLP6* showed enhanced tolerance to salinity stress [45]. Thus, we hypothesized that RcbHLH59 could potentially affect *PR*s and participate in salinity stress tolerance. To test this hypothesis, we screened and selected 232 *PR*s from the rose salinity stress transcriptome (GEO accession number PRJNA42884). Among them, 34 *PR*s were found to be significantly upregulated under salinity stress and were classified into eight categories: PR1, PR2, PR3, PR4, PR5, PR6, PR10, and PR12 (Supplementary Data Fig. S4). We then extracted and performed *cis*-regulatory element (CRE) analysis of these *PR*s upstream of 2000-bp promoter regions. As a result, we found that all genes contained a number of variations of bHLH-binding elements, such as CREs of the E-box (5′-CANNTG-3′) or G-box (5′-CACGTG-3′) (Supplementary Data Fig. S5). Based on this, we selected 13 PRs (Supplementary Data Table S3), which contained more variations in the bHLH binding sites of the G-box and E-box CREs, for further RT–qPCR analysis in both TRV-*RcbHLH59* and *RcbHLH59*-OE plants. As shown in Fig. 4, the expression profiles of four *PR*s (*RcPR4/1*, *RcPR5/1*, *RcPR6/1*, and *RcPR12/1*) were significantly upregulated in *RcbHLH59*-OE compared with those in the VC controls, among which the *RcPR4/1* gene was upregulated by 9.2-fold. No significant changes were observed in the expression of the other genes. In addition, the expression levels of four genes (*RcPR3/1*, *RcPR3/2*, *RcPR4/1*, and *RcPR5/1*) in TRV-*RcbHLH59* were found to be downregulated by >50% compared with those in the TRV controls. The expression levels of four genes (*RcPR1/1*, *RcPR4/2*, *RcPR6/1*, and *RcPR12/1*) were also slightly downregulated. Other genes (*RcPR2/1*, *RcPR2/2*, *RcPR6/2*, *RcPR10/1*, and *RcPR10/2*) showed no significant changes. In conclusion, these results indicate that only two genes (*RcPR4/1* and *RcPR5/1*) were upregulated in *RcbHLH59*-OE and downregulated in TRV-*RcbHLH59*. Notably, RcbHLH59 may positively regulate *RcPR4/1* and *RcPR5/1* in rose plants.

### RcbHLH59 directly binds to the *RcPR4/1* and *RcPR5/1* promoters

**Figure 5:**
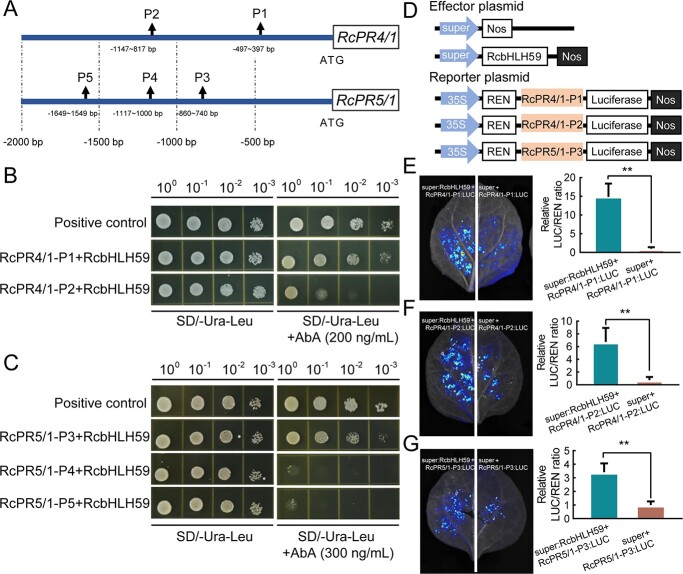
RcbHLH59 directly regulates RcPR4/1 and RcPR5/1. (A) Diagram of the truncated fragments of the promoters of *RcPR4/1* and *RcPR5/1*. Two fragments of *RcPR4/1* (P1 and P2) and three fragments of *RcPR5/1* (P3, P4, and P5) were selected. Arrows indicate the positions of the selected promoter fragments. (B) Y1H analysis of interaction between RcbHLH59 and RcPR4/1. Yeast strains co-transformed with pGADT7-RcbHLH59 (RcbHLH59) and RcPR4/1 promoters (RcPR4/1-P1 and RcPR4/1-P2) were grown on SD/−Ura/−Leu and SD−Ura/−Leu + 200 ng/ml aureobasidin A (AbA) medium for 3 days. (C) Y1H analysis of the interaction between RcbHLH59 and RcPR5/1. Yeast strains co-transformed with pGADT7-RcbHLH59 (RcbHLH59) and RcPR5/1 promoters (RcPR5/1-P3, RcPR5/1-P4, and RcPR5/1-P5) were grown on SD medium lacking Ura and Leu in the absence or presence of 300 ng/ml AbA for 3 days. pAbAi-p53 served as a positive control. 10^0^, 10^−1^, 10^−2^ and 10^−3^ represent the original concentration (OD_600_ = 0.2) and 10-, 100-, and 1000-fold dilutions of yeast, respectively. (D) Schematic of the effector and reporter vectors. The effector was generated by recombining the *RcbHLH59* gene into the pCAMBIA 1300 vector. The promoter fragments of RcPR4/1 (RcPR4/1-P1 and RcPR4/1-P2) and RcPR5/1 (RcPR5/1-P3) were cloned into the pGreenII 0800-LUC vector to generate reporter constructs. (E–G) Dual-luciferase analysis in tobacco leaves showed that RcbHLH59 activates the promoters of (E) RcPR4/1-P1, (F) RcPR4/1-P2, and (G) RcPR5/1-P3. Relative LUC/REN activity assays were used to verify that RcbHLH59 activates the promoters of RcPR4/1-P1, RcPR4/1-P2, and RcPR5/1-P3. Data are mean with standard deviation (*n* = 3) of at least three independent experiments. Statistically significant differences (^**^, *P* ≤ .01) were determined by *t*-tests.

As RcbHLH59 positively regulates the expression of *RcPR4/1* and *RcPR5/1*, we hypothesized that RcbHLH59 directly activates the expression of *RcPR4/1* and *RcPR5/1*. bHLH TFs function by recognizing the CREs of the E-box or G-box of their putative target gene promoter regions [46]. To test this hypothesis, we first analyzed the CREs of the *RcPR4/1* and *RcPR5/1* promoter regions. Interestingly, two and three bHLH-binding sites were found in the 2000-bp promoters of *RcPR4/1* and *RcPR5/1*, respectively (Supplementary Data Fig. S6). Next, we used a yeast one-hybrid (Y1H) approach to determine whether RcbHLH59 can bind to the promoters of these two modulation genes. Five fragments, including two (P1 and P2) of *RcPR4/1* and three (P3, P4, and P5) of *RcPR5/1*, covering the promoter regions were selected, wherein all fragments contained one or two E-box motifs (Fig. 5A). Next, we analyzed the self-activation of *RcPR4/1* and *RcPR5/1* in yeast cells (Supplementary Data Fig. S7) and constructed the Y1H assays. All yeast strains grew well in SD media lacking uracil (Ura) and leucine (Leu), whereas the yeast co-transformed with RcPR4/1-P1 + RcbHLH59, RcPR4/1-P2 + RcbHLH59, and RcPR5/1-P3 + RcbHLH59 exhibited normal growth in SD media lacking Ura and Leu and in the absence of 200 ng/ml (RcPR4/1) or 300 mg/ml (RcPR5/1) aureobasidin A (AbA) (Fig. 5B and C).

We then performed an *in vivo* luciferase activation assay to further explore the binding of RcbHLH59 to the *RcPR4/1* and *RcPR5/1* promoters. RcbHLH59 was cloned into the pCAMBIA1300 vector (super:RcbHLH59) as an expression vector. The promoter fragments of RcPR4/1-P1, RcPR4/1-P2, and RcPR5/1-P3 were fused to the firefly luciferase reporter gene (*LUC*), transformed into *Agrobacterium* GV3101, and then transiently transformed into *Nicotiana benthamiana* leaves. Firefly luciferase complementary imaging analysis showed that the tobacco surfaces co-transformed with super:RcbHLH59, wherein RcPR4/1-P1:LUC, RcPR4/1-P2:LUC, and RcPR5/1-P3:LUC were significantly luminescent compared with tobacco surfaces without super:RcbHLH59 (Fig. 5D–G). The quantitative results also provided supportive evidence for this. Thus, these results indicate that RcbHLH59 binds to the *RcPR4/1* and *RcPR5/1* promoters to activate *LUC* expression in *N. benthamiana* leaves. Collectively, these findings suggest that RcbHLH59 binds to the *RcPR4/1* and *RcPR5/1* promoters.

### *RcPR4/1* and *RcPR5/1* exert positive roles in salinity tolerance

To verify the effects of *RcPR4/1* and *RcPR5/1* under salinity stress, we first analyzed the transcript levels of *RcPR4/1* and *RcPR5/1* in the leaves and roots of rose plants under salinity stress (Supplementary Data Fig. S8). The results showed that the expression levels of *RcPR4/1* and *RcPR5/1* were similar to those of *RcbHLH59*. The transcripts of *RcPR4/1* and *RcPR5/1* peaked at 12 and 6 hours, respectively, in the roots and then decreased (Supplementary Data Fig. S8A and C). The *RcPR4/1* and *RcPR5/1* expression levels in leaves gradually increased with time and reached a peak at 48 hours (Supplementary Data Fig. S8B and D). It is worth noting that both *RcPR4/1* and *RcPR5/1* were significantly upregulated under salinity stress, and the transcripts of *RcPR4/1* and *RcPR5/1* in leaves were upregulated by 340-fold and 9-fold at 48 hours, respectively, compared with 0 hours.

**Figure 6:**
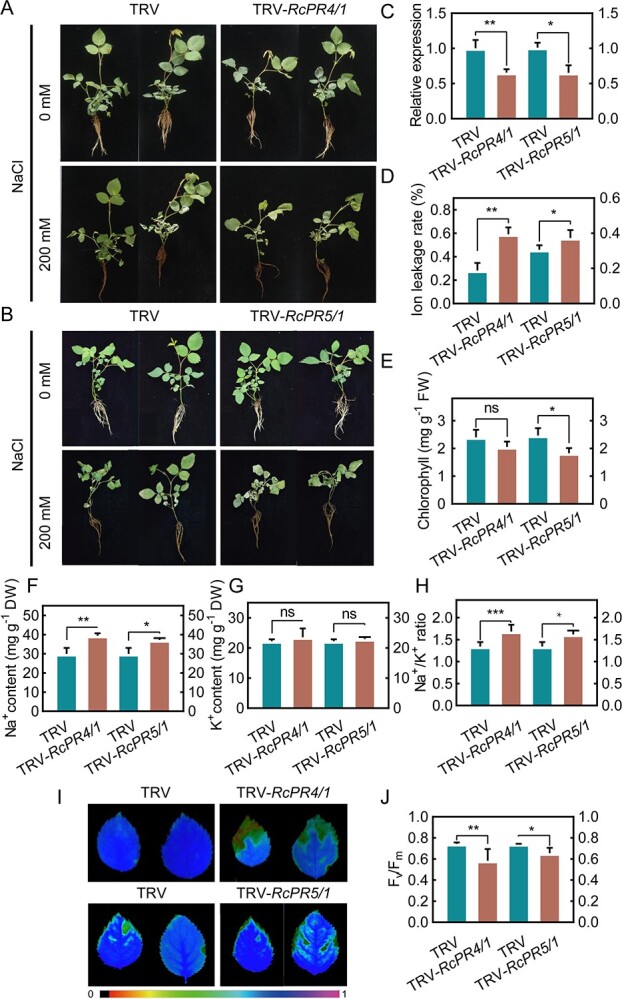
Silencing *RcPR4/1* or *RcPR5/1* decreases salt tolerance in rose. (A, B) Phenotypes of (A) TRV and TRV-*RcPR4/1* and (B) TRV and TRV-*RcPR5/1* rose plants under 200 mM NaCl for 3 days. (C) Relative expression of *RcPR4/1* and *RcPR5/1* in TRV, TRV-*RcPR4/1*, and TRV-*RcPR5/1* plants. *RcUBI2* was used as the internal control. (D) Ion leakage rate, (E) chlorophyll content, (F) Na^+^ content, (G) K^+^ content, and (H) Na^+^/K^+^ ratio in leaves of TRV, TRV-*RcPR4/1*, and TRV-*RcPR5/1* plants after 200 mM NaCl treatment. (I) Chlorophyll imaging and (J) quantification of leaves of TRV, TRV-*RcPR4/1*, and TRV-*RcPR5/1*. Error bars represent values from minimum to maximum. Data are mean with standard deviation (*n* = 3) of at least three independent experiments, each with more than three plants. Statistically significant differences (ns, *P* > .05; ^*^, *P* ≤ .05; ^**^, *P* ≤ .01; ^***^, *P* ≤ 0.01) were determined by *t*-tests.

We used the VIGS approach to silence *RcPR4/1* and *RcPR5/1* in rose plants under salinity stress (Fig. 6A and B). The expression of *RcPR4/1* and *RcPR5/1* was found to be reduced in both TRV-*RcPR4/1* (0.65-fold) and TRV-*RcPR5/1* (0.47-fold) compared with the TRV controls (Fig. 6C). The ion leakage rate was higher in both TRV-*RcPR4/1* and TRV-*RcPR5/1* than in the TRV controls (Fig. 6D). By contrast, the chlorophyll content was significantly decreased (0.67-fold) in TRV-*RcPR5/1* but remained almost unchanged in TRV-*RcPR4/1* (2.30 and 1.95 mg/g, respectively) (Fig. 6E).

We also examined the Na^+^ and K^+^ content and Na^+^/K^+^ ratio of silenced plants treated with 200 mM NaCl. The Na^+^ content in TRV-*RcPR4/1* and TRV-*RcPR5/1* was 39.4 and 37.1 mg/g, respectively, which was significantly higher than in the TRV controls (Fig. 6F). However, no significant difference was observed in the K^+^ content (Fig. 6G). Consistent with the Na^+^ and K^+^ contents, the Na^+^/K^+^ ratio was higher in both TRV-*RcPR4/1* (1.69) and TRV-*RcPR5/1* (1.61) compared with the TRV controls (Fig. 6H). Finally, we monitored the PSII of TRV, TRV-*RcPR4/1*, and TRV-*RcPR5/1* (Fig. 6I). As shown in Fig. 6J, the *F*_v_/*F*_m_ values of both TRV-*RcPR4/1* and TRV-*RcPR5/1* were lower than those of the TRV controls. Collectively, these results indicate that both *RcPR4/1* and *RcPR5/1* play positive roles in salinity stress in rose plants.

### *RcPR4/1* and *RcPR5/1* affect dynamic changes in callose accumulation and degradation

**Figure 7:**
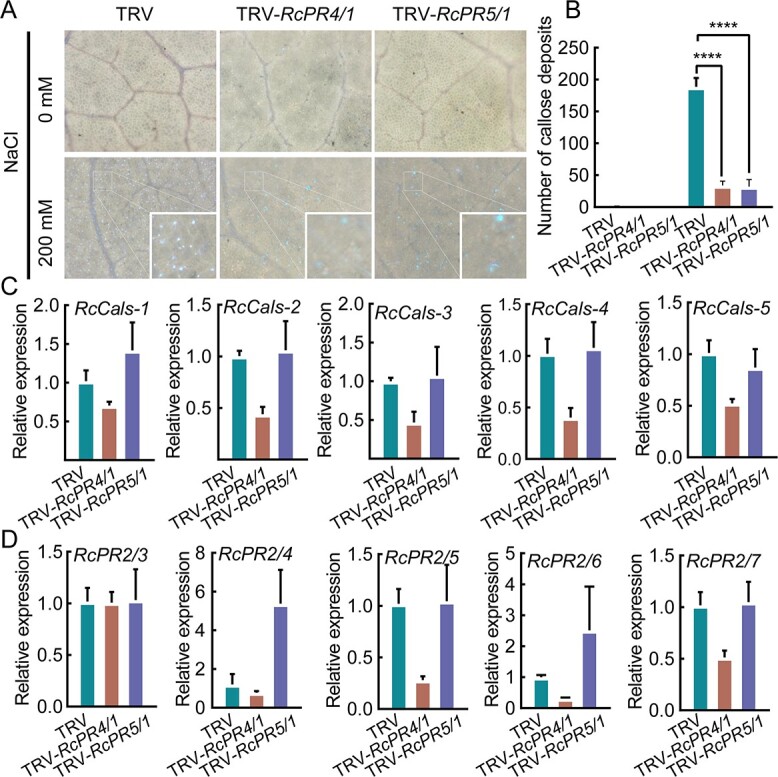
*RcPR4/1* and *RcPR5/1* influence the accumulation of corpus callosum. (A) Epidermis of plant leaves under a fluorescence microscope, with blue–white spots of callosum. The white border is the insets with photographic enlargements. (B) Statistical results of the number of corpus callosums. Error bars indicate standard deviations based on four biological replicates. Statistically significant differences (^****^, *P* ≤ .0001) were determined by one-way ANOVA (non-parametric or mixed). (C) Expression analysis of five *Cals* genes (*RcCals-1*, *RcCals-2*, *RcCals-3*, *RcCals-4*, and *RcCals-5*) and (D) five *PR2* genes (*RcPR2/3*, *RcPR2/4*, *RcPR2/5*, *RcPR2/6*, and *RcPR2/7*) in leaves of TRV, TRV-*RcPR4/1*, and TRV-*RcPR5/1* under salinity stress for 3 days. Error bars indicate standard deviations based on six biological replicates.

Given that PRs have been shown to mediate callose biosynthesis and degradation [27], we hypothesized that RcPR4/1 and RcPR5/1 could potentially affect callose composition under salinity stress. To test this hypothesis, we measured the callose content in TRV, TRV-*RcPR4/1*, and TRV-*RcPR5/1* under normal and 200 mM NaCl conditions. In the absence of NaCl, few corpus callosums were observed in the TRV, TRV-*RcPR4/1*, and TRV-*RcPR5/1* plants. However, under the 200 mM NaCl treatment, different degrees of corpus callosum accumulation were observed (Fig. 7A). In particular, the amount of callosum accumulation in TRV-*RcPR4/1* and TRV-*RcPR5/1* plants was much lower than that in TRV. The quantitative results also showed that the number of callose deposits was markedly lower in the leaves of both TRV-*RcPR4/1* and TRV-*RcPR5/1* compared with the TRV controls (Fig. 7B). These results suggest that *RcPR4/1* and *RcPR5/1* influence accumulation of the corpus callosum under salinity stress.

It is well known that callosum is synthesized by *callose synthase* (*Cals*) [39], while the *pathogenesis related genes* (*PR2*) inhibit callosum accumulation [40]. Based on this, we analyzed the expression levels of five *Cals* genes (*RcCals-1*, *RcCals-2*, *RcCals-3*, *RcCals-4*, and *RcCals-5*) and five *PR2* genes (*RcPR2/3*, *RcPR2/4*, *RcPR2/5*, *RcPR2/6*, and *RcPR2/7*) in both TRV-*RcPR4/1* and TRV-*RcPR5/1* (Fig. 7C and D). The results showed that the *Cals* genes were downregulated to varying degrees in TRV-*RcPR4/1* relative to TRV. However, this effect was not pronounced in TRV-*RcPR5/1*. For *PR2* genes, no significant changes were observed in *RcPR2/1* expression in TRV-*RcPR4/1*, whereas the other four *PR2* genes (*RcPR2/4, RcPR2/5, RcPR2/6*, and *RcPR2/7*) were significantly downregulated. For TRV-*RcPR5/1*, the expression levels of the three *PR2* genes *RcPR2/3*, *RcPR2/4*, and *RcPR2/7* did not change significantly, and two *PR2* genes (*RcPR2/4* and *RcPR2/6*) were significantly upregulated. This suggests that the expression of certain *Cals* and *PR2* genes are affected by *RcPR4/1* and *RcPR5/1*, which in turn affects callosum accumulation.

## Discussion

bHLHs are one of the most abundant TF families in plants and have been shown to play critical roles in the response to abiotic stress. The role of bHLHs under salinity stress has been studied widely in model and non-model plants, such as *A. thaliana* [47], *O. sativa* [19], *M. domestica* [48], *Solanum lycopersicum* [49], and *Myrothamnus flabellifolia* [50]. However, little is known about its role in roses. In this study we demonstrated that RcbHLH59, which is closely related to *Arabidopsis* AtbHLH59, plays a positive role in salinity stress tolerance. *Arabidopsis AtbHLH59* is induced by salinity stress, participates in physiological processes, such as Na^+^/K^+^ balance, redox, and transpiration rate, and improves plant tolerance to salinity stress [47]. Our data are consistent with these results, which demonstrated that silencing *RcbHLH59* reduces the salt tolerance of plants (Fig. 2), whereas the overexpression of *RcbHLH59* significantly improves salt tolerance (Fig. 3). In addition, *RcbHLH59* binds to the promoters of *RcPR4/1* and *RcPR5/1* to regulate the accumulation of callose, thereby promoting Na^+^/K^+^ balance, which in turn enhances salinity stress tolerance. This highlights RcbHLH59 as a key regulator and reveals for the first time the role of the RcbHLH59-RcPRs module in enhancing salt tolerance via the Na^+^/K^+^ balance through callose deposition.

Changes in the levels of ROS are essential for abiotic stress responses in plants [51]. An excessive accumulation of ROS induces crosslinks, base modifications, or deletions, as well as distorting genome stability and activating programmed cell death [52]. In fact, the accumulation of H_2_O_2_ and O_2_^−^ is often used to measure the degree of plant damage. Previous studies have found that tobacco plants overexpressing *RcMYBPA2* have less excess H_2_O_2_ and O_2_^−^ accumulation, suggesting that they have an improved tolerance of oxidative stress [53]. Furthermore, the increase in ROS levels is related to the control of stomatal pore size, the reduction of CO_2_ levels, and photosynthesis [54]. Therefore, the more serious the damage to the photosynthetic system, the more ROS accumulates in the plant. In this study, *RcbHLH59-*silenced plants were found to contain higher concentrations of H_2_O_2_ and O_2_^−^, indicating that TRV-*RcbHLH59* plants accumulated more ROS and suffered more damage (Fig. 3E and F). We performed DAB and NBT staining and observed less speckle accumulation in the leaves of *RcbHLH59-*OE plants than the controls (Fig. 3D), implying that *RcbHLH59* plays a positive role in salinity tolerance. These findings correlate well with those of tobacco *NtbHLH123* [55] and *M. flabellifolia MfbHLH38* [56], which have been reported to improve salt tolerance by scavenging ROS. Previous studies have shown that bHLHs confer abiotic stress tolerance by regulating photosynthesis [57]. In the present study, the *F*_v_/*F*_m_ value of TRV-*RcbHLH59* was found to be markedly lower than that of the control group under salinity stress (Supplementary Data Fig. S3), while *RcbHLH59*-OE had the opposite result (Fig. 3E and F). A higher *F*_v_/*F*_m_ was associated with *RcbHLH59* overexpression, indicating that *RcbHLH59* endowed plants with an enhanced tolerance of salt stress. These results show that the silenced plants had stronger photoinhibition, whereas the overexpressing plants had weaker photoinhibition and more adaptability.

Ionic stress is one of the most important components of salinity, caused by excessive Na^+^ accumulation and a disturbance in K^+^ homeostasis. In fact, maintaining the balance of the Na^+^/K^+^ ratio has become a key mechanism in response to salinity stress [58]. Many TFs are involved in this process, including the bHLH TF RIF1 (also named AIF2), and its partner RSA1 (SHORT ROOT IN SALT MEDIUM 1) in *Arabidopsis* [59]. The roots and leaves of tomatoes overexpressing *SlMYB102* have been reported to accumulate more K^+^ and less Na^+^, maintaining a better Na^+^/K^+^ ratio [9]. The overexpression of *SbbHLH85* disturbs the balance between Na^+^ and K^+^, thereby increasing salt tolerance in *Arabidopsis* [12]. Interestingly, a different phenomenon was observed in the present study. Here, the Na^+^ content in the leaves of TRV-*RcbHLH59* was found to be almost twice that of TRV. However, the K^+^ content in the leaves of TRV-*RcbHLH59* did not change significantly (Fig. 2G–I). These results suggest that *RcbHLH59* may function more specifically for Na^+^ than for K^+^.

PRs have a broad range of functions and are activated under abiotic and biotic stresses, including disease states, drought, saline conditions, and excessive cold or heat [28, 30]. In our study, two *PR*s (*RcPR4/1* and *RcPR5/1*) were screened in TRV-*RcbHLH59* and *RcbHLH59-*OE plants. These two genes were found to be downregulated in TRV-*RcbHLH59* cells and significantly upregulated in *RcbHLH59-*OE cells, indicating that *RcbHLH59* may regulate *RcPR4/1* and *RcPR5/1*. In addition, both *RcPR4/1* and *RcPR5/1* were significantly induced by salinity stress, indicating that they participated in tolerance of salinity stress. Previous studies have shown that *PR*s are regulated by Di19 and MYB TFs [33, 60]; however, *PR*s have not been reported to be regulated by bHLH. To verify whether RcbHLH59 regulated RcPR4/1 and RcPR5/1, Y1H experiments were performed, which demonstrated that RcbHLH59 binds to RcPR4/1-P1, RcPR4/1-P2, and RcPR5/1-P3. This was also confirmed by a dual-luciferase assay (Fig. 5). Furthermore, we validated the functions of *RcPR4/1* and *RcPR5/1* under salinity stress conditions. Similar to *RcbHLH59*, the silencing of *RcPR4/1* or *RcPR5/1* was found to affect the Na^+^/K^+^ balance and the photosynthetic system of plants, reducing their salt tolerance.

The PR family is known to play a major role in pathogen invasion [61]. For example, PR4 is a chitinase that degrades chitin and inhibits fungal growth, thereby improving plant disease resistance [62]. PR5 is a thaumatin-like protein (TLP) that also has antifungal activity and accumulates in plant cell wall adherents to resist pathogen invasion [63]. However, the details of the molecular mechanisms underlying PRs in response to abiotic stresses, such as the physiological effects of PR4 and PR5, remain poorly understood. In the present study, we focused on a polysaccharide named callose, which is similar in composition to cellulose, is synthesized under the influence of injury, cold, and various pathogens, and provides mechanical support for the plasma membrane or cell wall, resisting different environmental stresses [33–35]. The β-1,3-glucanase PR2 family has an inhibitory effect on callose synthesis [37]. Callose deposition is a defense response that can be regulated by many TFs, such as WRKYs [64]. Numerous studies have shown that the PR4 family exhibits synergistic effects with the PR2 family in response to abiotic and biotic stresses [65, 66]. Therefore, we believe that the PR2, PR4, and PR5 families may directly or indirectly affect callose accumulation and degradation. In this study, callose accumulation was found to be much lower in both TRV-*RcPR4/1* and TRV-*RcPR5/1* plants than in the control plants, indicating that *RcPR4/1* and *RcPR5/1* are involved in callose accumulation, providing enhanced tolerance of salinity.

Furthermore, expression of both *Cals* and *PR2* was found to be significantly downregulated in TRV-*RcPR4/1*, implying that RcPR4/1 not only promotes callose synthesis, but also influences callose degradation. In TRV-*RcPR5/1*, although the expression of *Cals* was not affected, the expression of two *PR2*s was significantly upregulated. An excessive accumulation of corpus callosum has been reported to affect permeability and hinder communication between cells [67]. The synthesis and degradation of calloses are relatively rapid reactions [68]. The breakdown of the corpus callosum can occur within a short time (5–10 minutes) of tissue injury [69]. Therefore, we speculated that a dynamic equilibrium relationship exists between the accumulation and decomposition of callose, and that *RcPR4/1*, a small-molecule protein, is abundant and plays a significant role in enhancing plant stress. In terms of *RcPR5/1*, although it has no effect on Cals, it had a synergistic inhibitory effect on PR2, indicating that *RcPR5/1* may function in the decomposition of callose.

Meanwhile, the Na^+^ content was found to be markedly higher in TRV-*RcPR4/1* and TRV-*RcPR5/1* than in the control group, although this did not hold for the K^+^ content. These results suggest that the accumulation of callose hinders the influx of Na^+^ and improves the Na^+^/K^+^ balance, which in turn increases tolerance to salinity stress. These findings are consistent with those of previous studies on exposure to heavy metal stress, which triggers changes in plasmodesmatal permeability through the breakdown of callose [70]. During heavy metal penetration, callose accumulates on the cell wall, and these changes can limit the penetration of heavy metals into cells and protect the plasma membrane and protoplasts from damage [36].

**Figure 8:**
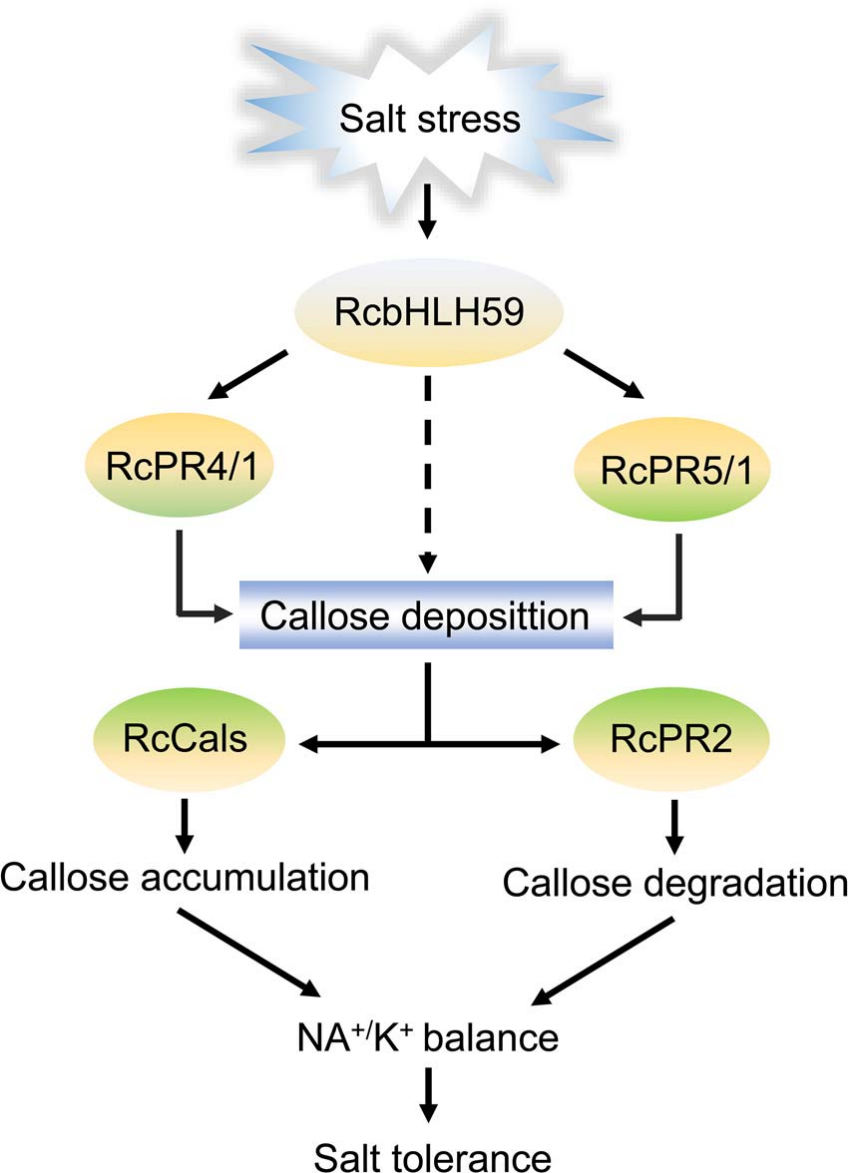
A working model of how RcbHLH59-RcPRs improves salt tolerance. Salinity stress induces transcriptional translation of RcbHLH59. It then binds to the promoters of *RcPR4/1* and *RcPR5/1*, influencing the expression of the *Cals* gene and *PR2* genes. This in turn promotes the accumulation of callosum and inhibits the degradation of callose, thus, improving the Na^+^/K^+^ balance and leading to tolerance to salinity stress.

In summary, our findings reveal how a novel RcbHLH59-RcPRs module promotes resistance to salinity stress in rose plants. As a new regulatory node, RcbHLH59 is linked to two major *PR*s: *RcPR4/1* and *RcPR5/1*. Under salinity stress, RcbHLH59 regulates *RcPR4/1*, thereby influencing *RcCals* expression and promoting callose accumulation. Additionally, RcbHLH59 also regulates *RcPR5/1*, leading to the inhibition of callose hydrolysis and improvement of the Na^+^/K^+^ balance, thereby enhancing salinity tolerance. RcbHLH59-RcPRs may act as a central component in response to salinity stress, allowing plants to balance the Na^+^/K^+^ ratio and callose deposition in response to salinity stress (Fig. 8). Overall, our findings highlight a novel molecular mechanism for the regulation of salinity stress tolerance and a possible strategy for improving the characteristics of rose plants through genetic engineering.

## Materials and methods

### Plant materials and growth conditions

Rose (*Rosa chinensis* ‘Old Blush’) tissue culture plants were propagated *in vitro*, as described above [42]. Rose stems with at least one node were grown on Murashige and Skoog (MS) base salts for ~45 days. The growth conditions were a temperature of 23 ± 1°C, light conditions of 16 hours light/ 8 hours dark, and a relative humidity of 50–60%. Next, the rose plants were removed from the MS and hydrocultured in 1/4 Hoagland solution. After 15 days of growth, plants with the same growth vigor were subjected to salt stress treatment with 0 (control) or 200 mM NaCl for 1, 3, 6, 12, 24, and 48 hours and used for further analysis. The NaCl concentration used was based on Bao *et al*. [71], and the experiments were conducted in a completely randomized design, each comprising three replicates.

Tobacco (*N. benthamiana*) leaves are easily infiltrated and are widely used in dual-luciferase assays [72] . Here the tobacco plants were grown in a mixed medium (vermiculite:nutritive soil, 1:1) and cultivated under controlled conditions (23 ± 1°C and a relative humidity of 50% with light conditions of 16 hours light/8 hours dark).

### Gene cloning and sequence analysis

Total RNA from rose leaves was extracted using the FastPure Plant Total RNA Isolation Kit (Vazyme Biotech, Nanjing, China), followed by reverse transcription using HiScript III RT SuperMix (Vazyme Biotech) to obtain cDNA. The coding sequence of *RcbHLH59* was amplified by PCR using the primers listed in Supplementary Data Table S1.

For sequence analysis, MEGA X [73] was used to create a neighbor-joining tree with 2000 bootstrap replicates. Eleven different plant species with bHLHs corresponding to abiotic stress were used*.* Detailed information on these proteins is provided in Supplementary Data Table S2. Multiple sequence alignment results were visualized using Jalview (http://www.jalview.org/getdown/release/). The pI and Mw were analyzed using ExPASy ProtParam (http://web.expasy.org/protparam/).

### Quantitative real-time PCR analysis

Total RNA was extracted from the roots and leaves of 9-week-old *R. chinensis* ‘Old Blush’ plants treated with 200 mM NaCl at six time points (0, 3, 6, 12, 24, and 48 hours). HiScript III All-in-one RT SuperMix Perfect for qPCR (Vazyme Biotech, Nanjing, China) was used to synthesize the cDNA. RT–qPCR was performed as previously described [45]. *RcUBI2* [74] was used as the internal control gene. Three biological replicates were performed for each treatment using the primers listed in Supplementary Data Table S1.

### Transcriptional activation analysis

For the transcriptional activation assay, the ORF of RcbHLH59 was amplified by PCR with specific primers (Supplementary Data Table S1) and fused to the pGBKT7 vector containing the GAL4 DNA-binding domain. The fusion vector pGBKT7-RcbHLH59 (BD-RcbHLH59), negative control, positive control, and empty vector were transformed into yeast strain Y2HGOLD (Weidi, Shanghai, China). The transformed yeast cells were then grown on SD/−Trp and SD/−Trp−His−Ade growth media with or without X-α-Gal. After growing for 3 days at 28°C in an incubator, the yeast colonies were observed and photographed.

### Virus-induced gene silencing

The VIGS method [42] was used to silence *RcbHLH59*, *RcPR4/1*, and *RcPR5/1*. First, 216-, 340-, and 420-bp fragments of the 5′ region of *RcbHLH59*, *RcPR4/1*, and *RcPR5/1* were used as silencing fragments. These fragments were cloned into the pTRV2 vector to generate pTRV2-*RcbHLH59*, pTRV2-*RcPR4/1*, and pTRV2-*RcPR5/1*. Next, pTRV2-*RcbHLH59*, pTRV2-*RcPR4/1*, pTRV2-*RcPR5/1*, and pTRV2, together with pTRV1, were transformed into *Agrobacterium tumefaciens* GV3101. Each transformant was cultured in LB medium supplemented with 10 mM 2-(N-morpholino)ethanesulfonic acid (MES), 40 μM acetosyringone (AS), 100 mg/l rifampicin, and 100 mg/l kanamycin. After overnight incubation (28°C, 200 rpm), the cell strains were centrifuged (5000 *g* for 8 minutes), resuspended in permeabilization buffer (10 mM MgCl_2_, 10 mM MES, and 200 μM AS), and adjusted to an OD_600_ of 2.0. Cells containing pTRV2-*RcbHLH59*, pTRV2-*RcPR4/1*, pTRV2-*RcPR5/1*, or pTRV2 were mixed with cells containing pTRV1 and incubated in the dark for 4 hours. Then, hydroponic rose plants of the same size cultured for ~15 days with the same growth performance were infiltrated with *A. tumefaciens* GV3101 cells containing plasmids TRV (negative control), TRV-*RcbHLH59*, TRV-*RcPR4/1*, and TRV-*RcPR5/1* in a vacuum of 0.7 atmospheres. Lastly, these were cultured at 8°C for 3 days in the dark. Following incubation, leaves were incubated in an aqueous environment with 0 or 200 mM NaCl under the same growth conditions.

### Transient overexpression in rose

*RcbHLH59* was transiently overexpressed according to a previous method [75] with some modifications. First, the ORF of *RcbHLH59* was cloned into a modified pCAMBIA 1300 vector to generate the *RcbHLH59* overexpression line *RcbHLH59*-OE. Overexpression samples were generated from the middle and lower leaves of the roses. *A. tumefaciens* GV3101 carrying pCAMBIA 1300 (VC) or *RcbHLH59*-OE was collected by centrifugation, resuspended in permeabilization buffer (10 mM MgCl_2_, 10 mM MES, and 200 μM AS), and adjusted to an OD_600_ of 1.0. Then, tender leaves from the same position at the top of the nutrient hydroponic rose plants of the same size were used for infiltration. Next, these were cultured at 24°C for 3 days in the dark. After incubation, both VC and *RcbHLH59*-OE leaves were infiltrated with 0 or 200 mM NaCl in a vacuum of 0.7 atmospheres, followed by culturing in the same concentrations of NaCl. The experiment was conducted in triplicate, each experiment containing at least six replicates.

### Physiological analysis

The chlorophyll content was determined as described previously [42]. Briefly, leaf samples were rinsed with distilled water and blotted dry with filter paper. Both sides of the main veins of the leaves were cut into filaments with a width of <1 mm, and samples of 0.2 g were weighed. The leaves were then immersed in 95% ethanol and placed in the dark for 3 days. The total chlorophyll content (mg/g) was calculated as follows: total chlorophyll content = (18.08 A_649_ + 6.63 A_649_)/100. The plant leaf imaging and parameters (*F*_v_/*F*_m_) were recorded using a chlorophyll fluorometer (IMAG-MAX/L; Walz, Germany). Each test condition included four biological and six technical replicates.

To determine the ion leakage rate, 0.2 g of leaf sample was rinsed with distilled water and blotted dry using filter paper. The sample was placed in a tube containing 10 ml of deionized water and soaked for 8–12 hours. Then, the liquid conductivity (*I*_1_) was measured using a conductivity meter. Subsequently, the plant leaves were boiled for 20–30 minutes, deionized water was added to make up to 10 ml, and the liquid conductivity (*I*_2_) was measured at the same position. Ion leakage was calculated using the following equation: ion leakage = (*I*_1_ – *I*_0_)/(*I*_2_ – *I*_0_), where I_0_ is the deionized water conductance rate.

To determine the contents of Na^+^ and K^+^, the samples were placed in an oven at 80°C. Next, samples of 0.2 g were weighed and placed in 50-ml tubes. The tube contents were mixed with 12 ml HNO_3_ and 2 ml HClO_4_ mixed acid and allowed to stand overnight. On the following day, the digestion tube was heated to a low boil (160°C) to evaporate the bulk of the acid. The solution was diluted to 25 ml with water for testing. Lastly, the Na^+^ and K^+^ contents of the liquid to be tested were measured using an optical emission spectrometer (PerkinElmer Instruments, USA).

Treated leaves (0.1 g) were used to determine the concentrations of H_2_O_2_ and O_2_^−^ using H_2_O_2_ and O_2_^−^ content detection kits (Solarbio, Beijing, China), respectively. Samples of VC and RcbHLH59-OE were immersed in 1 mg/ml DAB or 1 mg/ml NBT staining solution. The samples were incubated in the dark overnight, decolorized in a boiling water bath for 10 minutes, and then photographed.

### Yeast one-hybrid assay

The Y1H was carried out using the Matchmaker^®^ Gold Yeast Two-Hybrid System (Takara Bio, USA) with some modifications. The full-length coding sequence of RcbHLH59 was ligated into the pGADT7 vector to generate prey constructs. The promoter sequences of RcPR4/1 (RcPR4/1-P1 and RcPR4/1-P2) and RcPR5/1 (RcPR5/1-P3, RcPR5/1-P4, and RcPR5/1-P5) were cloned into the pAbAi vector to generate the bait constructs. The constructed pAbAi vector was digested with BstBI and co-transformed into Y1HGLOD yeast along with pGADT7-RcbHLH59. After validating the sequence, four successive dilutions (10^0^, 10^−1^, 10^−2^, and 10^−3^) of spots were prepared and plated onto SD/−Ura/−Leu and SD/−Ura/−Leu plates supplemented with or without 200 or 300 ng/ml AbA, respectively.

### Luciferase reporter assay

The coding sequence of RcbHLH59 was cloned into pCAMBIA 1300 to synthesize super RcbHLH59 as an effector plasmid. The promoter sequences of RcPR4/1 and RcPR5/1 were cloned into the pGreenII 0800-LUC vector to generate the reporter constructs. The two plasmids were then transformed into the GV3101 strain and used to infect the back of tobacco leaves. After storing in the dark for 3 days, D-luciferin sodium salt (Sangon Biotech, Shanghai, China) was sprayed on the leaves. Fluorescence was observed using a living plant fluorescence detector (Vilber, France) and quantified using a dual-luciferase reporter reagent (Vazyme Biotech, Nanjing, China).

### Callose staining

Leaves from TRV, TRV-*RcPR4/1*, and TRV-*RcPR5/1* plants (with or without 200 mM NaCl) were stained with aniline blue staining solution (Solarbio, Beijing, China) for 1 hour in the dark. Samples with green fluorescence were considered calluses and observed under a Carl Zeiss Axio Scope A1 microscope. The number of calluses was counted using the ImageJ software (https://imagej.nih.gov/ij/).

### Statistical analysis

All statistical analyses were performed using SPSS v25.0 (SPSS Inc., Chicago, IL, USA). Data were statistically analyzed using Student’s *t*-test or one-way analysis of variance (ANOVA), followed by a least significant difference (LSD) test. Statistical analyses of independent experiments are reported as the mean ± standard deviation.

## Acknowledgements

This research was supported by the National Key Research and Development Program (2018YFD1000400) and National Natural Science Foundation of China (Grant No. 32002084). We would like to thank Editage (www.editage.cn) for English language editing.

## Author contributions

X.J. and L.S. designed and performed the experiments. Y.Z., S.Y., L.G., and S.L. conducted the experiments and analyzed the data. X.J., L.O., and L.S. wrote and revised the manuscript.

## Data availability

Sequence data in this study can be found in the Genome Database for Rosaceae (https://www.rosaceae.org/) and the accession numbers are listed 804 in Supplementary Data Table S3.

## Conflict of interest

The authors declare they have no competing interests.

## Accession numbers

Sequence data in this study can be found in the Genome Database for Rosaceae (https://www.rosaceae.org/) and the accession numbers are listed in Supplementary Data Table S3.

## Supplementary data

Supplementary data is available at *Horticulture Research* online.

## Supplementary Material

Web_Material_uhac291
