# Supplementary material for: RcbHLH59-RcPRs module enhances salinity stress tolerance by balancing Na+/K+ through callose deposition in rose (Rosa chinensis)
Source: Hortic Res. 2022 Dec 30;10(3):uhac291. doi: 10.1093/hr/uhac291 (PMC10018784; doi:10.1093/hr/uhac291)
Supplement: Web_Material_uhac291 [file web_material_uhac291.zip › Supplementary results-1209.docx]

**Rose (*Rosa chinensis*) RcbHLH59-RcPRs module enhances salinity stress tolerance by balancing Na^+^/K^+^ through callose deposition**


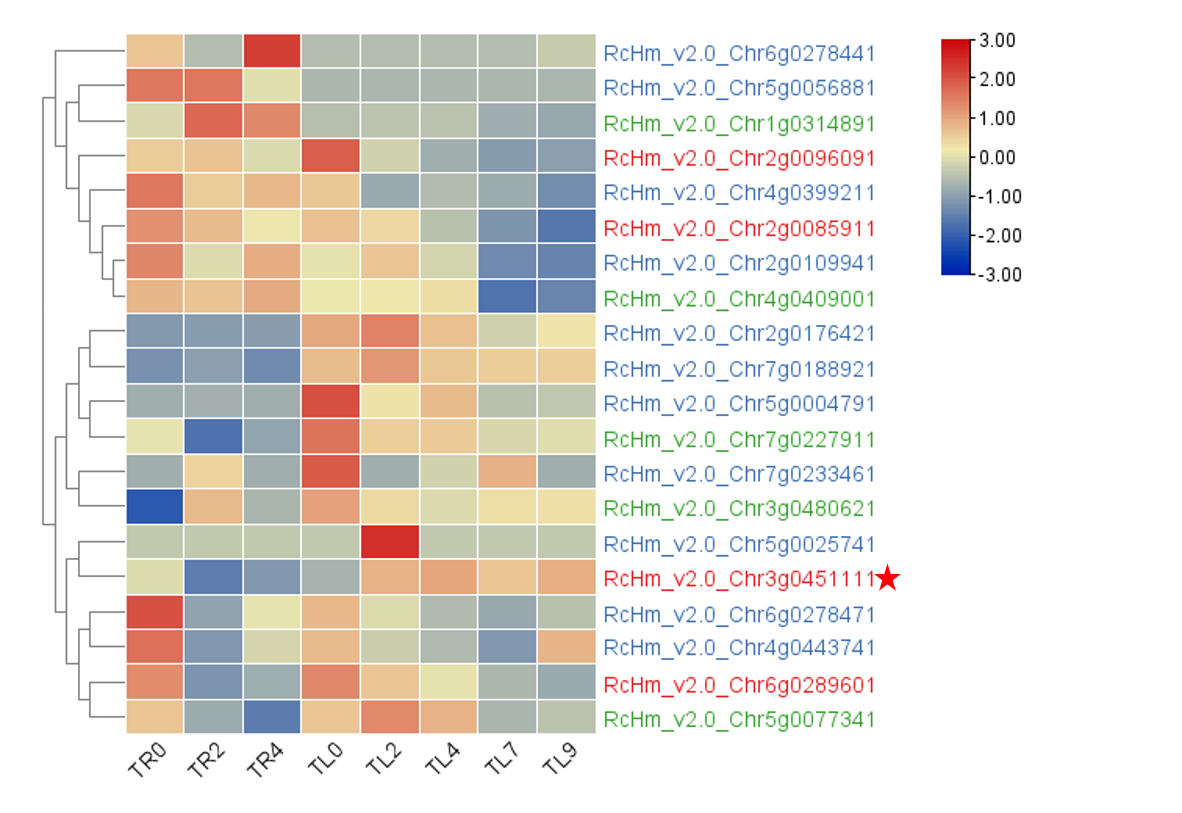


**Figure S1** Expression profiles of RcbHLHs under salt treatment.

Expression heatmap of three groups of RcbHLHs under salt stress. The red genes represent bHLH family XⅠ group; green genes represent bHLH family I X group; blue genes represent bHLH family XII group. The red five-pointed star indicates RcbHLH59.


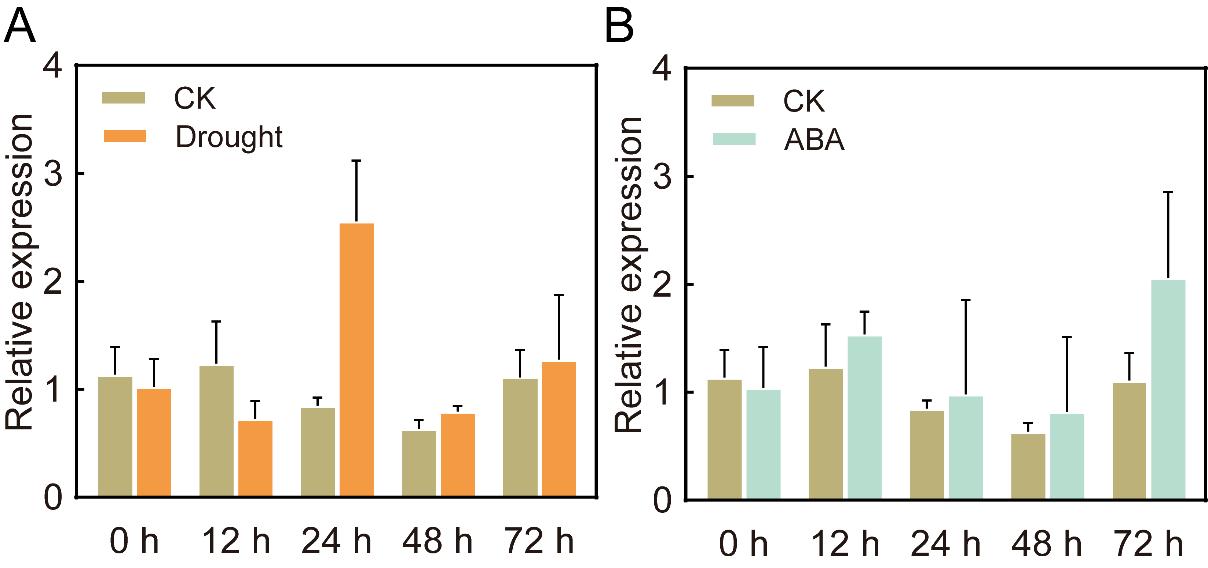


**Figure S2** Expression of *RcbHLH59* under drought and ABA stress conditions.

Expression profiles of RcbHLH59 in rose leaves under (A) 20% PEG 6000 and (B) 100 μM ABA. Error bars indicate standard deviations (SDs) based on three biological replicates.


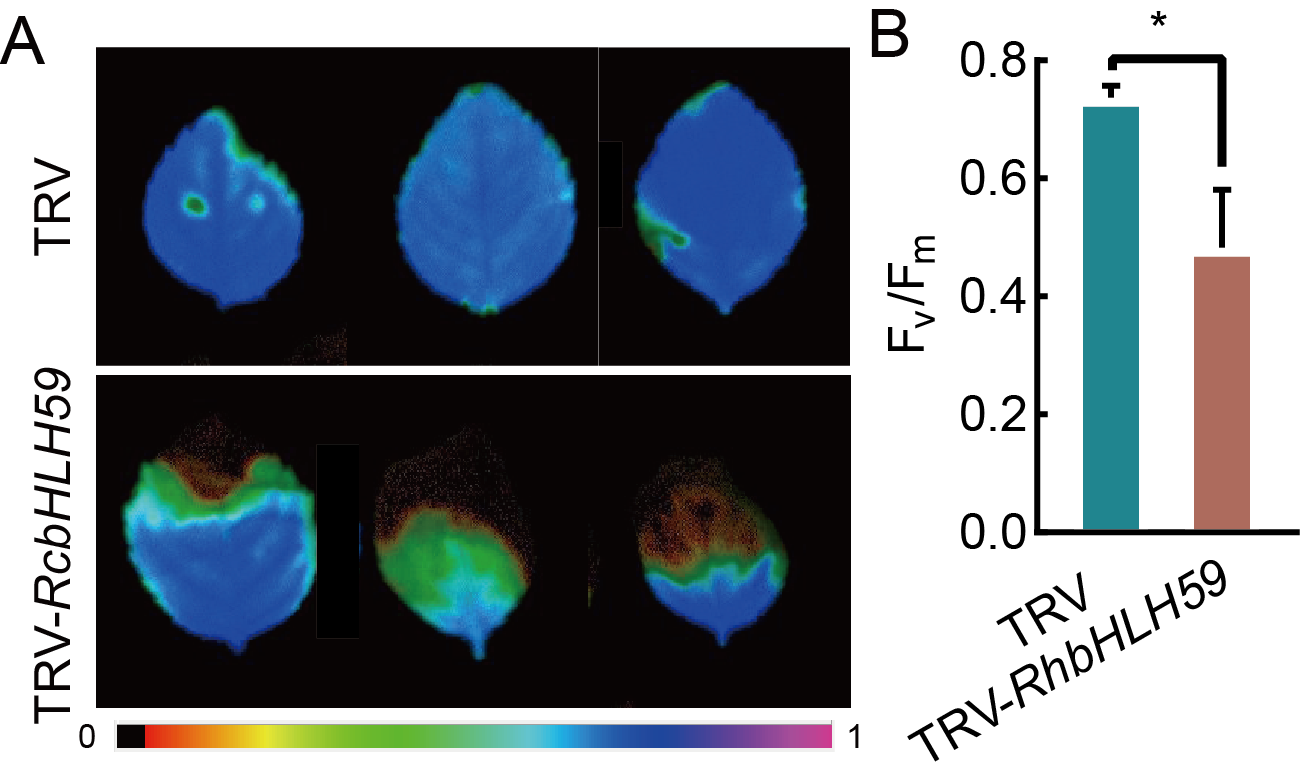


**Figure S3** Silencing of *RcbHLH59* affects the photosynthetic system of rose leaves.

(A) Chlorophyll fluorescence images of rose leaves of TRV and TRV-*RcbHLH59* under 200 mM NaCl treatment. Error bars at the bottom represent the values from minimum to maximum. (B) Quantification of Chlorophyll Imaging. Data are mean with SD (n=3) of at least three independent experiments.


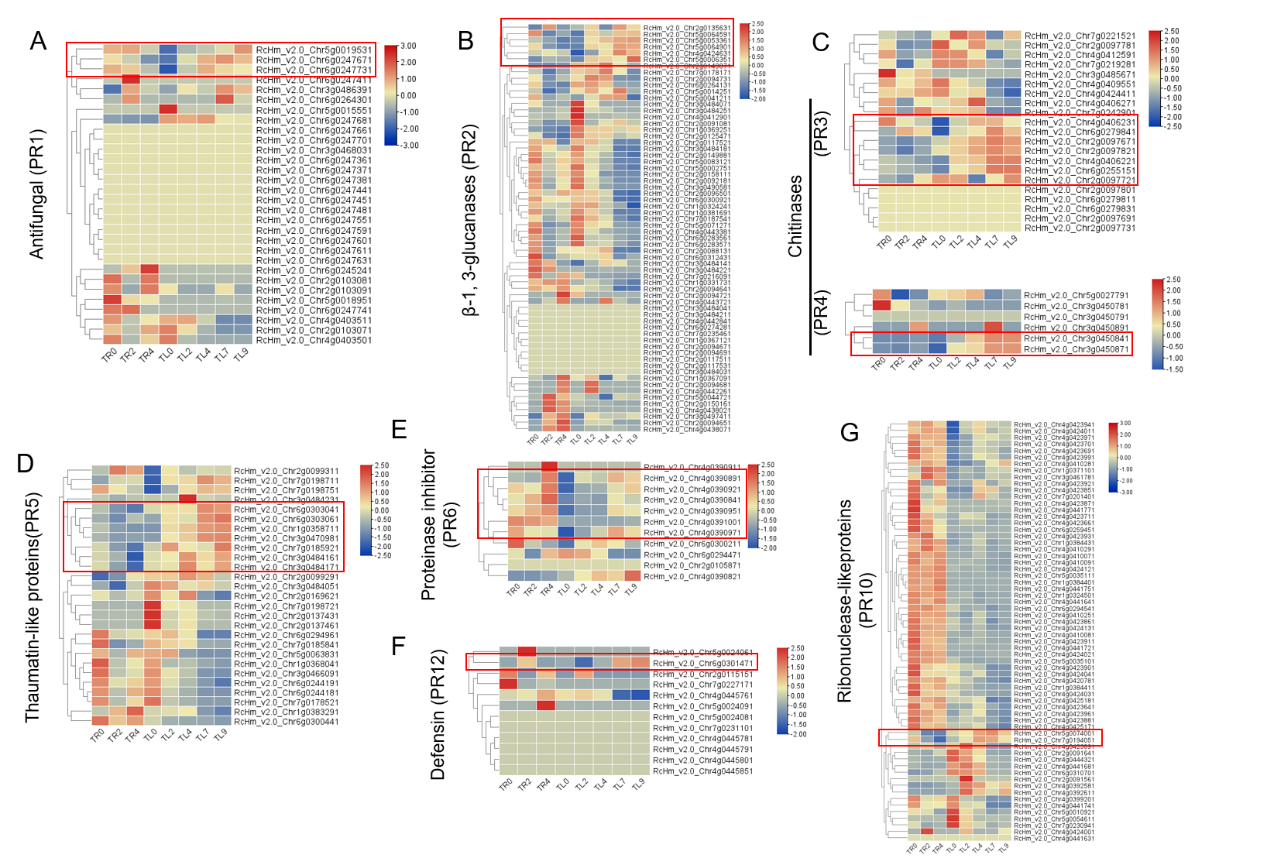


**Figure S4** Expression pattern profiles of RcPRs under salt stress treatments.

Expression heatmap of PRs under salt stress, including (A) Antifungal (PR1), (B) β-1,3-Glucanase (PR2), (C) Chitinase (PR3 and PR4), (D) Thaumatin-like (PR5), (E) Proteinase inhibitor (PR6), (G) Ribonuclease-like (PR10) and (F) Defensin (PR12). Red boxes indicate PRs with the same expression pattern as *RcbHLH59* under salt stress. Salt stress treatment were conducted 1:1:3 ratio of 0.4% mmol/L NaCl: Na_2_SO_4_: NaHCO_3_ in roots with 0 days (TR0), 2 days (TR2), 4 days (TR4), in leaves with 0 days (TL0), 2 days (TL2), 4 days (TL4), 7 days (TL7), and 9 days (TL9), respectively. The data from salt stress (Tian et al.,2018) were used.

**
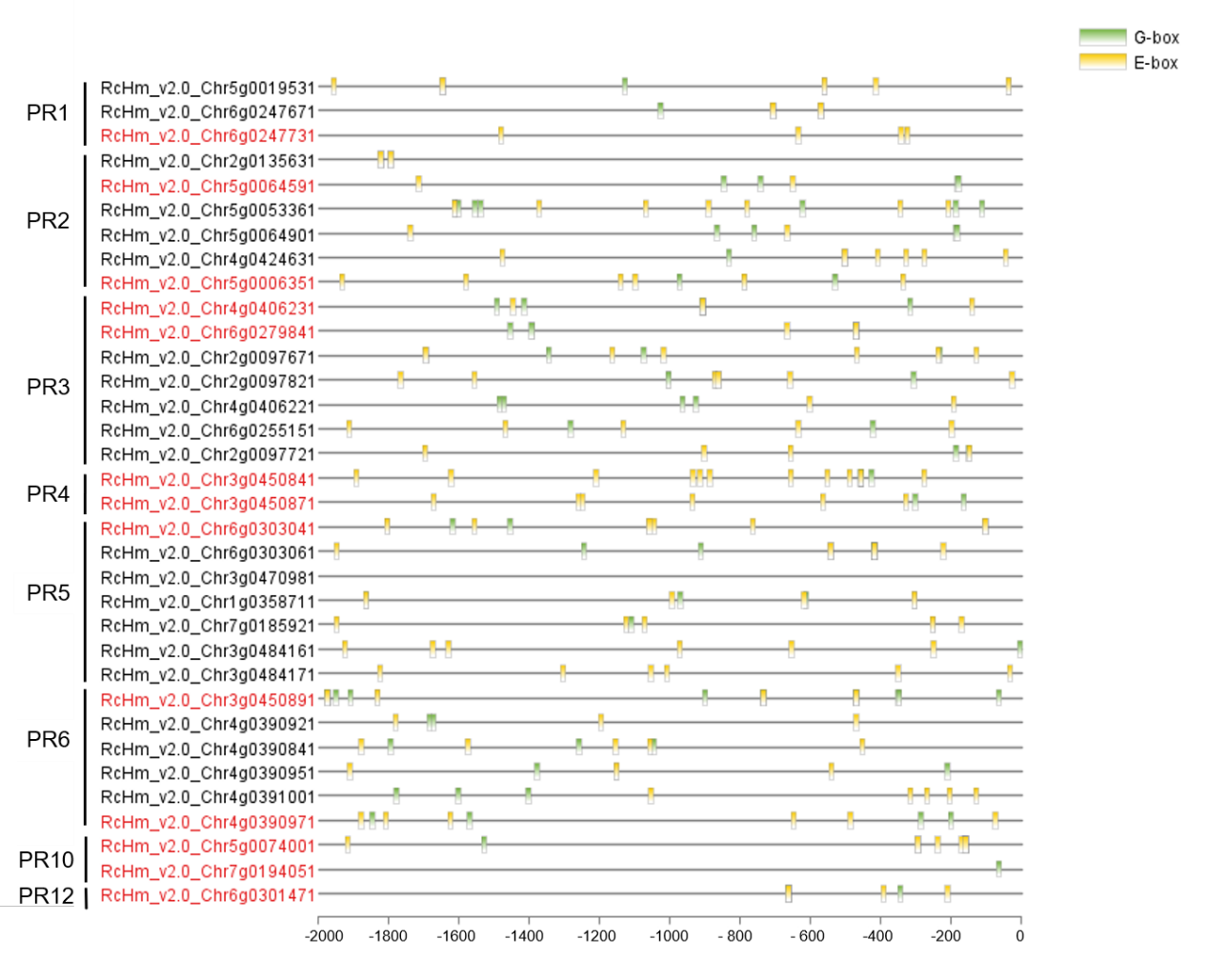
**

**Figure S5** Analysis of E-box and G-box in 2,000 bp promoters of 34 PRs.

Number of E-box (5' CANNTG 3') or G-box (5' CACGTG 3') cis-acting elements (CEs) in the promoter region of PRs (2 kb upstream of the translation start site). Green boxes represent G-boxes and yellow boxes represent E-boxes. Red gene name Indicates genes with more E-box or G-box in each type of PRs.


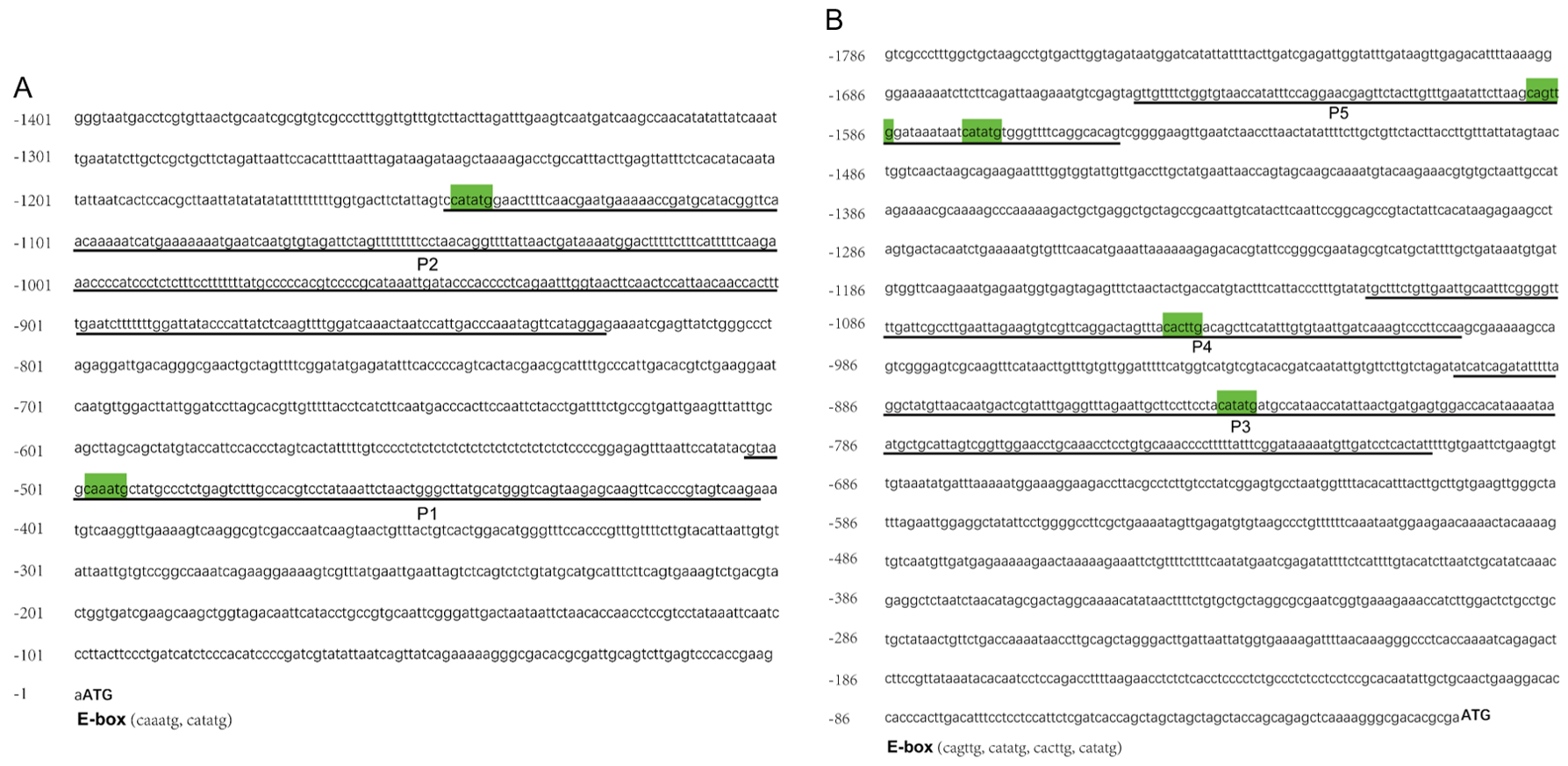


**Figure S6** Distribution of bHLH-binding sequences in the promoters of *RcPR4/1* and *RcPR5/1*.

(A) RcPR4/1 and (B) RcPR5/1 promoter sequence distribution. The green sequence indicates where the E-box exists. Black line segments represent clipped segments.


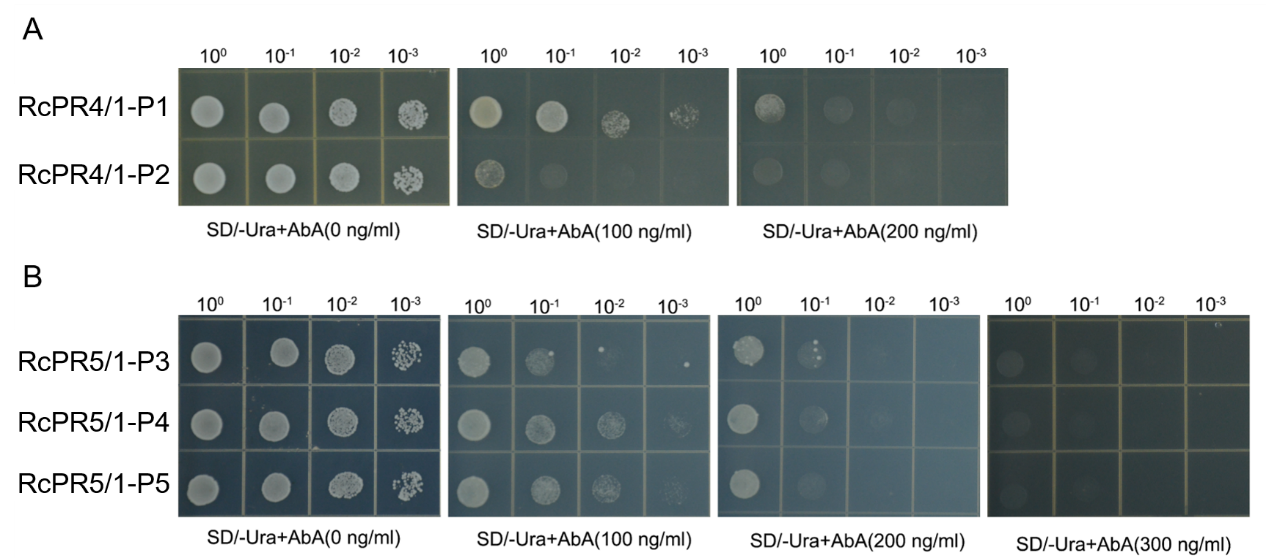


**Figure S7** Self-activation detection of RcPR4/1 and RcPR5/1 in yeast cells.

(A) The promoters of *RcPR4/1* (P1 and P2), and (B) the promoters of *RcPR5/1* (P3, P4, and P5) were constructed into the pAbAi vector and grown on SD/-Ura/-Leu with different concentrations of Aureobasidin A (AbA) to verify the self-activation phenomenon. 10^0^, 10^-1^, 10^-2^ and 10^-3^ represent the original concentration (OD_600_=0.2), 10-fold, 100-fold and 1000-fold dilutions of yeast, respectively.


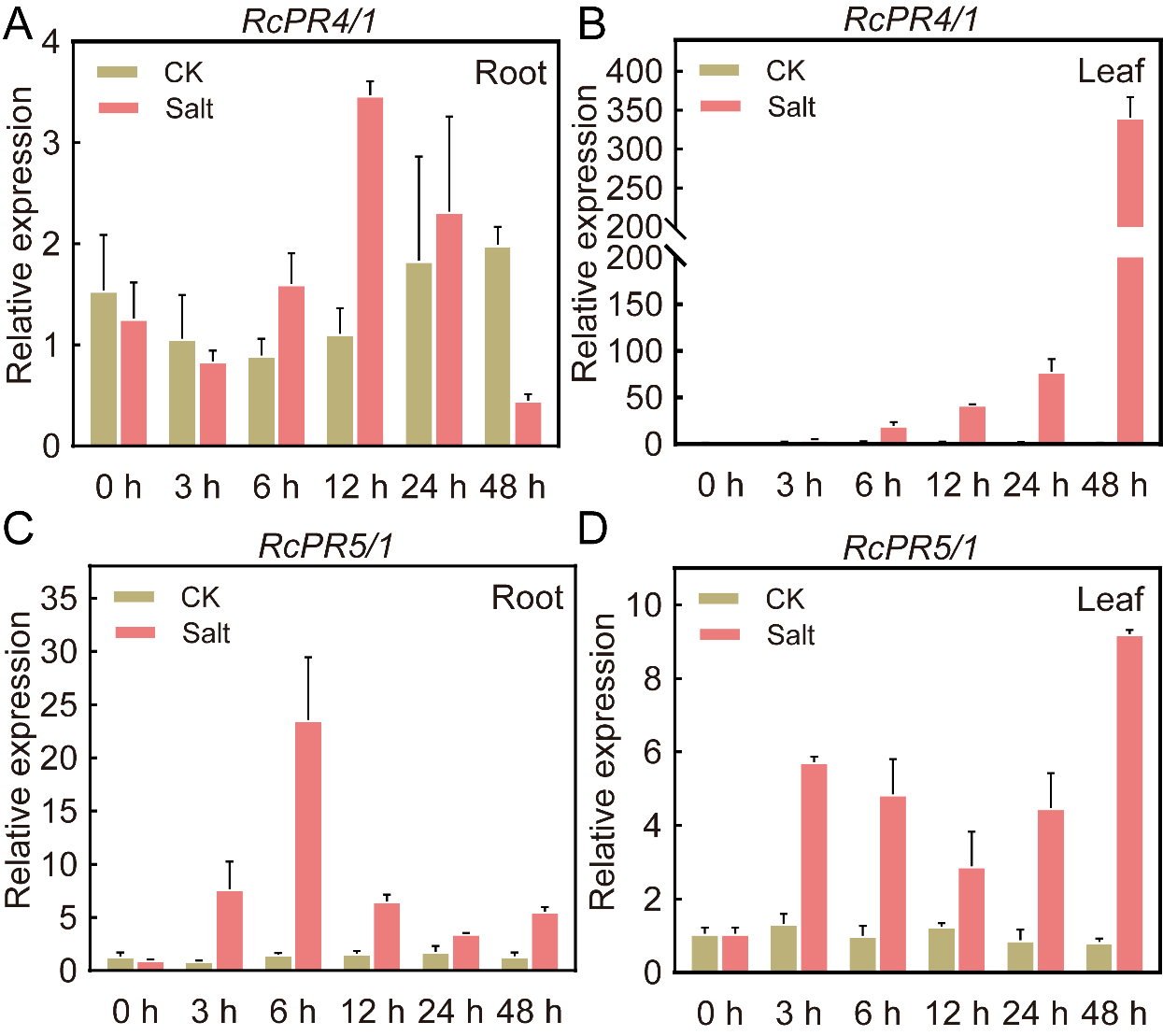


**Figure S8** Expression of *RcPR4/1* and *RcPR5/1* in rose roots and leaves under salt stress.

Expression profiles of *RcPR4/1* in rose (A) roots, and (B) leaves under 200 mM NaCl treatment. Expression profiles of *RcPR5/1* in rose (C) roots, and (D) leaves under 200 mM NaCl treatment. Error bars indicate standard deviations (SDs) based on three biological replicates.

**Table S1** The primers sequences used in this study

| **Name** | **Forward** | **Reverse** |
| --- | --- | --- |
| For gene cloning in rose | | |
| *RcbHLH59* | ATGGCGGGCAATCCGC | CTAGGAAGAGTTGTGTGATTCCGG |
| For construction of silencing expression vector | | |
| TRV-*RcbHLH59* | gtgagtaaggttacc*gaattc*ATGGCGGGCAATCCGCCT | gagacgcgt*gagctc*ggtaccCCAACCGACCCGACTCCT |
| TRV-*RcPR4* | cgactctag*tctaga*aagcttATGGCCGGGAAACAATGC | catggtacc*ggatcc*actagtGTCCCCACAGTCGACAAAGTCG |
| TRV-RcPR5 | cct*ccatgg*ggatccggtaccATGGCTCCTCCATTAACACCC | ggcctcgagacgcgtgagctcAGGGAGGTTGTAACCGTCGAC |
| For construction of overexpression vector | | |
| *RcbHLH59*-OE | cgactctagtctaga*aagctt*ATGGCGGGCAATCCGCCT | cactagtatttaaat*gtcgac*CTAGGAAGAGTTGTGTGATTCCGG |
| *RcPR4/1*-OE | cgactctagtctaga*aagctt*ATGGCCGGGAAACAATGC | cactagtatttaaat*gtcgac*TTAGTCCCCACAGTCGACAAAGT |
| *RcPR5/1*-OE | cgactctagtctaga*aagctt*ATGGCTCCTCCATTAACACCC | cactagtatttaaat*gtcgac*TTAGAAGAGGTGCTGCCACAACT |
| For yeast vector construction in rose | | |
| Pro-*RcPR5/1* | TGGCTGCTAAGCCTGTGACTTG | TTGAGCTCTGCTGGTAGCTAGCTG |
| *Pro-RhPR4*/1 | TGTTGAACGCATATTGGTG | TTTCTGATAACTGATTAATATACGA |
| pAbAi-RcPR4/1-P1 | ccaaagcttgaattc*gagctc*CGTAAGCAAATGCTATGCCCT | agcacatgcctcgag*gtcgac*TCTTGACTACGGGTGAACTTGCT |
| pAbAi -RcPR4/1-P2 | ccaaagcttgaattc*gagctc*CCATATGGAACTTTTCAACGAATG | agcacatgcctcgag*gtcgac*CTCCTATGAACTATTTGGGTCAATGG |
| pAbAi -RcPR5/1-P3 | ccaaagcttgaattc*gagctc*ATCATCAGATATTTTTAGGCTATGTTAACA | agcacatgcctcgag*gtcgac*AATAGTGAGGATCAACATTTTTATCCG |
| pAbAi -RcPR5/1-P4 | ccaaagcttgaattc*gagctc*TGCTTTCTGTTGAATTGCAATTTC | agcacatgcctcgag*gtcgac*TGGAAGGGACTTTGATCAATTACA |
| pAbAi -RcPR5/1-P5 | ccaaagcttgaattc*gagctc*GTTGTTTTCTGGTGTAACCATATTTCC | agcacatgcctcgag*gtcgac*CTGTGCCTGAAAACCCACATATG |
| For dual luciferase vector construction in rose | | |
| RcPR4/1-P2:LUC | gtcgacggtatcgat*aagctt*CGTAAGCAAATGCTATGCCCT | agtggatcccccggg*ctgcag*TCTTGACTACGGGTGAACTTGCT |
| RcPR4/1-P3:LUC | gtcgacggtatcgat*aagctt*CCATATGGAACTTTTCAACGAATG | agtggatcccccggg*ctgcag*CTCCTATGAACTATTTGGGTCAATGG |
| RcPR5/1-P4:LUC | gtcgacggtatcgat*aagctt*ATCATCAGATATTTTTAGGCTATGTTAACA | agtggatcccccggg*ctgcag*AATAGTGAGGATCAACATTTTTATCCG |
| For RT-qPCR in rose | | |
| qRT-*PR12/1* | ATGGCTTTGATTCGTTTATTTCTCT | CTTGGTGTACTTCAGAGCGTAG |
| qRT-*PR10/1* | ATGCTAGATCCTGTGGATGTG | ATCAGCACATAATCCGCC |
| qRT-*PR10/1* | ATGGACGCGGTGGTGA | GGAGCCGCGCTTCTT |
| qRT-*PR1/1* | ATGGCTTTGATTCGTTTATTTC | CTTGGTGTACTTCAGAGCGTAG |
| qRT-*PR2/1* | ATGCTAGCTGCATTTGCAA | CAAGTCGGTTGCATCCAC |
| qRT-*PR2/2* | ATGGAGCCGAGGAAGCTC | GAAGATCTTGACGCGGTCG |
| qRT-*PR3/1* | ATGTTGTACTTGGCCGTGTC | TTGGCTTTGGCAACCG |
| qRT-*PR3/2* | ATGGCTTTCCAAGTTACAATTAG | AGGACCTTCTTTACAACCCTTG |
| qRT-*PR4/1* | ATGGCCGGAAAACAATGC | GGGCTTGTCGGCATCC |
| qRT-*PR4/2* | ATGCGTCGTGTTTTTGGTG | TTCCAAGGGCTTGTCGG |
| qRT-*PR5/1* | ATGGCTCCTCCATTAACACC | TTTCTGGAGGGCGAAGC |
| qRT-*PR6/1* | ATGTCCGATCAATGCGAAG | ATCTGTATCGACCCAAACACG |
| qRT-*PR6/2* | ATGGGTTCAGAATGTGAAGGTAA | TTAACCGATAATCGGAGTTTTTA |
| qRT-*RcCals-1* | ATGGCATATAGAAGGGGCTTT | TTTCTCAAATGCATAGAAGCGA |
| qRT-*RcCals-2* | ATGTCGTCGTCGAGAGCG | GTGGGATCCAACCTATGAGCTT |
| qRT-*RcCals-3* | ATGGCTCAAGTTGTGGATCG | TAACCATGCTCACATAGGATTCTA |
| qRT-*RcCals-4* | ATGGCTAGGGTTTACGACAATT | GCCTGCTCACACAGAATTCTG |
| qRT-*RcCals-5* | ATGTTGTCCTGCTATTTCACCAC | TTGCACTACAGACTGAGAAGCAA |
| qRT-*RcPR2/3* | ATGGGAAAGGCAAATATACTTGC | AGCTCCTTGGTTAGGATCATAGAG |
| qRT-*RcPR2/4* | ATGGAGCCTTCTAGCTTCTGTT | GAACGCCTTCAGCACCTT |
| qRT-*RcPR2/5* | ATGGCAGCGTTGCAGTCAC | GAGTTGAGTGATCGGTAGAGGA |
| qRT-*RcPR2/6* | ATGGCTATGAGATCTGTTCAGAG | CTTTCTCATTCTCGGCGCTT |
| qRT-*RcPR2/7* | ATGGCCGATTCCTCAGTTACT | CATGTGGAAACCCAGCGG |

**Table S2** List of GenBank accession numbers for 19 genes used in Fig.1

| **Gene name** | **Gene ID** | **References** |
| --- | --- | --- |
| *RcbHLH59* | RcHm_v2.0_Chr3g0451111 |  |
| *ZmbHLH124* | GRMZM2G132550 | Wei et al., 2021 |
| *AtbHLH59* | At4g02590 | He et al., 2022 |
| *AtbHLH106* | AT2G41130 | Ahmad et al., 2015 |
| *MdbHLH130* | MDP0000581816 | Zhao et al., 2020 |
| *PebHLH35* | KJ363186.1 | Dong et al., 2014 |
| *CsbHLH041* | Solyc07g039570.2.1 | Li et al., 2020 |
| *AtbHLH122* | At1G51140 | Liu et al., 2014 |
| *AtbHLH92* | At5g43650 | Jiang et al., 2009 |
| *AmDEL* | AAA32663.1 | Wang et al., 2016 |
| *VabHLH1* | JQ911779.1 | Xu et al., 2014 |
| *FtbHLH2* | AMK74868.1 | Yao et al., 2018 |
| *SmbHLH001* |  | Pires et al., 2010 |
| *OsbHLH012* | LOC_Os01g39480 | Pires et al., 2010 |
| *OsbHLH025* | LOC_Os01g09990 | Pires et al., 2010 |
| *AtbHLH017* | At2g46510 | Singh et al., 2013 |
| *AtbHLH082* | At5g58010 | Pires et al., 2010 |
| *AtbHLH134* | At5g15160 | Butt et al., 2017 |
| *AtbHLH003* | At4g16430 | Pires et al., 2010 |

| Wei S, Xia R, Chen C et al. *ZmbHLH124* identified in maize recombinant inbred lines contributes to drought tolerance in crops. Plant Biotechnol J. 2021; **19**:2069-2081. |
| --- |
| He Z, Wang Z, Nie X et al. UNFERTILIZED EMBRYO SAC 12 phosphorylation plays a crucial role in conferring salt tolerance. Plant Physiol. 2022;**188**:1385-1401. |
| Ahmad A, Niwa Y, Goto et al. bHLH106 Integrates Functions of Multiple Genes through Their G-Box to Confer Salt Tolerance on *Arabidopsis*. PLoS One. 2015; **10**:e0126872-e0126894. |
| Zhao Q, Fan Z, Qiu L et al. *MdbHLH130*, an Apple bHLH Transcription Factor, Confers Water Stress Resistance by Regulating Stomatal Closure and ROS Homeostasis in Transgenic Tobacco. Front Plant Sci. 2020; **11**:543696. |
| Dong Y, Wang C, Han X et al. A novel bHLH transcription factor PebHLH35 from *Populus euphratica* confers drought tolerance through regulating stomatal development, photosynthesis and growth in *Arabidopsis*.Biochem Bioph Res Co . 2014; **450**: 453-458. |
| Li J, Wang T, Han J et al. Genome-wide identification and characterization of cucumber *bHLH* family genes and the functional characterization of *CsbHLH041* in NaCl and ABA tolerance in *Arabidopsis* and cucumber. BMC Plant Biol. 2020; 20:272. |
| Liu W, Tai H, Li S et al. *bHLH122* is important for drought and osmotic stress resistance in *Arabidopsis* and in the repression of ABA catabolism. New Phytol. 2014;201:1192-1204. |
| Jiang Y, Yang B, Deyholos MK. Functional characterization of the Arabidopsis *bHLH92* transcription factor in abiotic stress. Mol Genet Genomics. 2009; 282:503-516. |
| Wang F, Zhu H, Kong W et al. The *Antirrhinum AmDEL* gene enhances flavonoids accumulation and salt and drought tolerance in transgenic *Arabidopsis*. Planta. 2016; 244:59-73. |
| Xu W, Zhang N, Jiao Y et al. The grapevine basic helix-loop-helix *(bHLH*) transcription factor positively modulates *CBF*-pathway and confers tolerance to cold-stress in *Arabidopsis*. Mol Biol Rep. 2014; 41:5329-5342. |
| Yao P, Sun Z, Li C et al. Overexpression of *Fagopyrum tataricum FtbHLH2* enhances tolerance to cold stress in transgenic Arabidopsis. Plant Physiol Biochem. 2018; 125:85-94. |
| Pires N, Dolan L. Origin and diversification of basic-helix-loop-helix proteins in plants. Mol Biol Evol. 2010; 2:862-74. |
| Li H, Sun J, Xu Y et al. The bHLH-type transcription factor AtAIB positively regulates ABA response in *Arabidopsis*. Plant Mol Biol. 2007; 65:655–665. |
| Lee S, Lee S, Yang K.Y et al. Overexpression of *PRE1* and its homologous genes activate Gibberellin-dependent responses in *Arabidopsis thaliana*. Plant Cell Physiol. 2006; 47:591–600. |

| **Table S3** List of accession numbers in this study | |
| --- | --- |
| **Gene name** | **Gene ID** |
| *RcbHLH59* | RcHm_v2.0_Chr3g0451111 |
| *RcPR4/1* | RcHm_v2.0_Chr3g0450871 |
| *RcPR5/1* | RcHm_v2.0_Chr6g0303041 |
| *RcPR12/1* | RcHm_v2.0_Chr6g0301471 |
| *RcPR10/1* | RcHm_v2.0_Chr5g0074001 |
| *RcPR10/2* | RcHm_v2.0_Chr7g0194051 |
| *RcPR1/1* | RcHm_v2.0_Chr6g0247731 |
| *RcPR2/1* | RcHm_v2.0_Chr5g0006351 |
| *RcPR2/2* | RcHm_v2.0_Chr5g0064591 |
| *RcPR3/1* | RcHm_v2.0_Chr6g0279841 |
| *RcPR3/2* | RcHm_v2.0_Chr4g0406231 |
| *RcPR4/2* | RcHm_v2.0_Chr3g0450841 |
| *RcPR6/1* | RcHm_v2.0_Chr4g0390891 |
| *RcPR6/2* | RcHm_v2.0_Chr4g0390971 |
| *RcCals-1* | RcHm_v2.0_Chr7g0191411 |
| *RcCals-2* | RcHm_v2.0_Chr1g0313341 |
| *RcCals-3* | RcHm_v2.0_Chr1g0313351 |
| *RcCals-4* | RcHm_v2.0_Chr5g0080661 |
| *RcCals-5* | RcHm_v2.0_Chr5g0080681 |
| *RcPR2/3* | RcHm_v2.0_Chr1g0331731 |
| *RcPR2/4* | RcHm_v2.0_Chr3g0484181 |
| *RcPR2/5* | RcHm_v2.0_Chr7g0187541 |
| *RcPR2/6* | RcHm_v2.0_Chr2g0094641 |
| *RcPR2-7* | RcHm_v2.0_Chr2g0125471 |
| *RcBUI2* | RcHm_v2.0_Chr1g0359561 |
